# Identification of DNA Methylation Changes in Newborns Related to Maternal Smoking during Pregnancy

**DOI:** 10.1289/ehp.1307892

**Published:** 2014-06-06

**Authors:** Christina A. Markunas, Zongli Xu, Sophia Harlid, Paul A. Wade, Rolv T. Lie, Jack A. Taylor, Allen J. Wilcox

**Affiliations:** 1 Epidemiology Branch, and; 2 Laboratory of Molecular Carcinogenesis, National Institute of Environmental Health Sciences, National Institutes of Health, Department of Health and Human Services, Research Triangle Park, North Carolina, USA; 3 Department of Global Public Health and Primary Care, University of Bergen, Bergen, Norway; 4 Medical Birth Registry of Norway, Norwegian Institute of Public Health, Bergen, Norway; * These authors contributed equally to this work.

## Abstract

Background: Maternal smoking during pregnancy is associated with significant infant morbidity and mortality, and may influence later disease risk. One mechanism by which smoking (and other environmental factors) might have long-lasting effects is through epigenetic modifications such as DNA methylation.

Objectives: We conducted an epigenome-wide association study (EWAS) investigating alterations in DNA methylation in infants exposed *in utero* to maternal tobacco smoke, using the Norway Facial Clefts Study.

Methods: The Illumina HumanMethylation450 BeadChip was used to assess DNA methylation in whole blood from 889 infants shortly after delivery. Of 889 mothers, 287 reported smoking—twice as many smokers as in any previous EWAS of maternal smoking. CpG sites related to maternal smoking during the first trimester were identified using robust linear regression.

Results: We identified 185 CpGs with altered methylation in infants of smokers at genome-wide significance (*q*-value < 0.05; mean Δβ = ± 2%). These correspond to 110 gene regions, of which 7 have been previously reported and 10 are newly confirmed using publicly available results. Among these 10, the most noteworthy are *FRMD4A*, *ATP9A*, *GALNT2*, and *MEG3*, implicated in processes related to nicotine dependence, smoking cessation, and placental and embryonic development.

Conclusions: Our study identified 10 genes with newly established links to maternal smoking. Further, we note differences between smoking-related methylation changes in newborns and adults, suggesting possible distinct effects of direct versus indirect tobacco smoke exposure as well as potential differences due to age. Further work would be needed to determine whether these small changes in DNA methylation are biologically or clinically relevant. The methylation changes identified in newborns may mediate the association between *in utero* maternal smoking exposure and later health outcomes.

Citation: Markunas CA, Xu Z, Harlid S, Wade PA, Lie RT, Taylor JA, Wilcox AJ. 2014. Identification of DNA methylation changes in newborns related to maternal smoking during pregnancy. Environ Health Perspect 122:1147–1153; http://dx.doi.org/10.1289/ehp.1307892

## Introduction

Cigarette smoke contains > 7,000 chemicals, of which hundreds are known to be harmful and at least 69 are known to cause cancer [Centers for Disease Control and Prevention (CDC) 2010]. Although the significant health effects of smoking are well recognized, smoking remains the largest preventable cause of death in the United States (CDC 2010). In addition to the deleterious direct effects on the person who smokes, smoking can also have indirect effects, for example, on the developing embryo or fetus. Maternal smoking during pregnancy is associated with substantial infant morbidity and mortality (Dietz et al. 2010). Although smoking is a modifiable risk behavior that influences the health of both the infant and the mother, only about 45% of women who report that they smoke 3 months before pregnancy quit during pregnancy (CDC 2012). Exposure to the fetus thus remains an important area of research, with little known about the molecular changes that occur in newborns in response to exposure *in utero*. Further, if these changes persist for an extended period of time (or are permanent), they could have implications for disease development later in life.

The “developmental origins of disease” hypothesis (Gluckman and Hanson 2004) proposes that environmental exposures occurring during development can cause biological changes that influence disease susceptibility later in life. One potential mechanism is through an alteration of the fetal epigenome, which may have long-term consequences (Waterland and Michels 2007). One of the most well-studied forms of epigenetic modification is DNA methylation, which occurs predominantly at position C5 of cytosine in cytosine–guanine dinucleotides (CpGs). DNA methylation plays an important role in human health and has been associated with a growing number of diseases including cancer, imprinting disorders, and repeat-instability diseases (Robertson 2005).

Several studies have examined DNA methylation changes associated with indirect maternal tobacco smoke exposure *in utero* [reviewed by Suter et al. (2013)]. These studies have examined changes in DNA from placenta, cord blood, buccal cells, or granulocytes by taking either a targeted (i.e., one or a few genes) or a more global approach (i.e., epigenome-wide). However, only one study to date has used a high-density DNA methylation array (the Illumina HumanMethylation450 BeadChip), to assess DNA methylation in newborns related to maternal smoking during pregnancy (Joubert et al. 2012).

To further explore DNA methylation changes in infants related to maternal smoking during pregnancy, we performed an epigenome-wide association study (EWAS) using whole blood from 889 infants in the Norway Facial Clefts Study (NCL), including 287 whose mothers smoked during the first trimester. As a replication set, we used publically available results [obtained from Supplemental, Table S1, in Joubert et al. (2012)] and considered two levels of replication: *a*) site level at the exact CpG and *b*) gene level for other CpG sites in or near the same gene. In addition, we considered the overlap between smoking-associated methylation in adults and newborns, with special focus on genes that may be unique to each.

## Methods

*Study population*. The present study is based on infants from the NCL. NCL is a national population-based case–control study of cleft lip and cleft palate, disorders characterized by the incomplete fusion of the lip and/or palate during development. The study design has been previously described in detail (Wilcox et al. 2007). Briefly, between the years 1996 and 2001, all families of newborns referred for cleft surgery in Norway were contacted; 88% of those eligible agreed to participate (*n* = 573). Controls were selected by a random sampling of roughly 4 per 1,000 live births in Norway during that same time period; 76% of those eligible agreed to participate (*n* = 763). NCL was approved by the Norwegian Data Inspectorate and Regional Medical Ethics Committee of Western Norway, and informed consent was provided by both the mother and father. In the current study, a subset of 418 facial cleft cases (lip only = 107, palate only = 144, lip and palate = 167) and 480 controls were selected based on DNA availability. Newborn blood was collected at the delivery hospital in plain glass capillary tubes by heel stick and then frozen and shipped to a central laboratory in Oslo for phenylketonuria and other routine genetic analysis. Collection was identical for cases and controls. Study population characteristics are shown in Tables 1 and 2.

**Table 1:** Characteristics of mothers in the study according to maternal smoking (*n* or mean ± SD).

Characteristic	Nonsmokers	Smokers	*p*-Value^*a*^
All mothers	602	287
Alcohol use^*b*^			4.7 × 10^–4^*
0	410	158
1–3	104	64
4–6	37	20
≥ 7	45	42
Education			2.7 × 10^–6^*
Less than high school	57	61
High school and above	545	226
Parity^*c*^			0.12
1	239	128
2	209	102
3	117	39
≥ 4	37	18
Age at delivery (years)	29.6 ± 4.9	28.0 ± 4.9	3.1 × 10^–6^*
Prepregnancy BMI (kg/m^2^)	23.6 ± 4.1	23.4 ± 4.3	0.56
Folic acid supplement (μg)^*d*^			0.02*
0	354	194
1–399	134	58
≥ 400	114	35
Dietary folate (μg)			0.18
0–171	163	88
172–214	150	56
215–264	123	65
≥ 265	136	54
Multivitamins^*d*^			0.36
No	391	196
Yes	211	91
^***a***^*p*-Values were calculated from Fisher’s exact test for categorical variables and Student’s *t*-test for numerical variables. ^***b***^Total number of drinks during the first trimester. ^***c***^Includes index child. ^***d***^During month before pregnancy and first 2 months of pregnancy. *Nominally associated (*p *< 0.05) with maternal smoking status during pregnancy (smokers vs. nonsmokers).			

**Table 2:** Characteristics of infants in the study according to maternal smoking (*n* or mean ± SD).

Characteristic	Nonsmokers	Smokers	*p*-Value^*a*^
All Infants	602	287
Sex			0.25
Female	252	132
Male	350	155
Facial cleft status			1.8 × 10^–4^*
Control	347	126
Cleft lip with or without cleft palate	160	113
Cleft palate only	95	48
Gestational age (weeks)	38.4 ± 7.7	37.2 ± 9.9	0.06
Birth weight (kg)	3.6 ± 0.6	3.4 ± 0.7	3.4 × 10^–6^*
^***a***^*p*-Values were calculated from Fisher’s exact test for categorical variables and Student’s *t*-test for numerical variables. *Nominally associated (*p *< 0.05) with maternal smoking status during pregnancy (smokers vs. nonsmokers).			

*Tobacco smoke exposure*. Information about maternal tobacco smoke exposure during pregnancy was obtained through mother’s questionnaire around 3–4 months after delivery. An English translation of this questionnaire can be found on the study’s website (National Institute of Environmental Health Sciences 2008). Mothers were asked about active smoking during the first trimester (average number of cigarettes smoked per day or per month) and passive smoke exposure at any time during the pregnancy (average number of hours per day she was within 2 m of a smoker). Environmental tobacco smoke exposure (passive smoking) *in utero* was not associated with DNA methylation in newborns [false discovery rate (FDR) *q* < 0.05], so we combined nonsmokers, passive (≥ 1 hr/day within 2 m of a smoker), and infrequent smokers (< 1 cigarette per day) into one group (*n* = 607). All active smokers were also combined (*n* = 290). We explored the possibility of a dose–response relationship between the extent of smoke exposure and the level of methylation at genome-wide significant CpG sites (FDR *q* < 0.05). Three exposure classes were defined for this analysis: *a*) 607 non-active smokers: nonsmokers (*n* = 437), passive (*n* = 131), and infrequent smokers (*n* = 39), *b*) 141 light smokers (1–5 cigarettes/day), and *c*) 149 moderate (6–10 cigarettes/day; *n* = 107) and heavy (≥ 11 cigarettes/day; *n* = 42) smokers. For a CpG site to meet our criteria for a dose–response relationship it must satisfy two requirements: *a*) a mean methylation level that progressively increases or decreases across the three categories of smoking, and *b*) the 3-level smoking variable must be significant (FDR *q* < 0.05) when treated as a continuous variable in the robust linear regression model. One mother used snuff tobacco; her baby was excluded from all analyses.

*Analysis*. A description of DNA methylation data generation (Illumina HumanMethylation450), quality assessment, and pre-processing (e.g., normalization) are provided (see Supplemental Material, Methods: Data generation, Data cleaning and quality assessment, and Data pre-processing). After exclusions (See Supplemental Material, Methods: Data cleaning and quality assessment), 889 samples (*n*_smokers_ = 287, *n*_nonsmokers_ = 602) and 357,320 CpG probes remained for analysis. A robust linear regression model was employed using the R package, MASS (Venables and Ripley 2002), to test the association between maternal smoking status (binary variable: active smokers versus nonsmokers and passive/infrequent smokers) and methylation (β-value) at each CpG site, adjusting for facial cleft status (control, cleft lip with or without cleft palate, and cleft palate only), infant’s sex, and two technical factors: batch effects (96-well plate) and bisulfite conversion efficiency (model 1) (see Supplemental Material, Methods, Data pre-processing). A sensitivity analysis was also conducted adjusting for several potential confounders. Maternal alcohol consumption, education, age, body mass index (BMI), dietary folate, folic acid supplement use, multivitamin use, parity, gestational age, and infant’s birth weight were all considered as possible confounders. Only those associated with maternal smoking (*p* < 0.05) (Tables 1 and 2) and that could potentially influence DNA methylation were added to the secondary model, referred to below as model 2. Cellular heterogeneity is a potential confounder if the distribution of blood cell subtypes differs by maternal smoking status. To correct for cellular heterogeneity, we implemented the method developed by Houseman et al. (2012) to estimate five different cell proportions for each sample and then adjust for those in our robust linear regression model (model 2). A reference data set [GSE35069 (Reinius et al. 2012)] was used first to identify DNA methylation profiles that were specific to each of the five cell subtypes. This information was then used to generate sample-specific cell proportion estimates that were included in model 2 to correct for five leukocyte subtype proportions (see Supplemental Material, Table S1, for a comparison between newborns exposed and unexposed). To correct for multiple testing, we estimated the FDR using the *q*-value framework (Storey and Tibshirani 2003).

## Results

**Figure 1:**
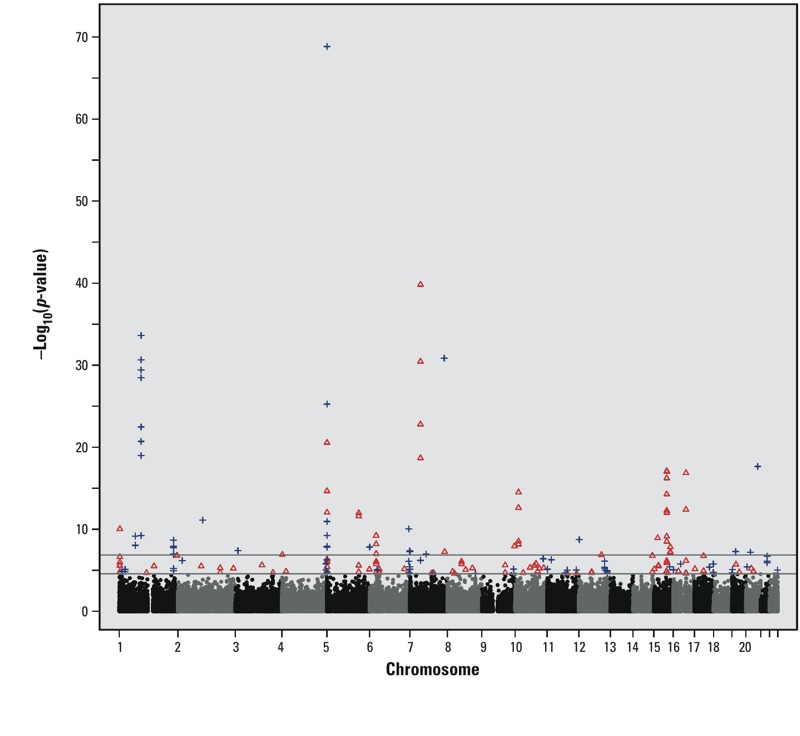
Manhattan plot of epigenome-wide results based on model 1. The lower gray horizontal line marks genome-wide significance using FDR *q *< 0.05 as a cutoff (*n *= 185 CpGs); the higher gray horizontal line marks genome-wide significance using a Bonferroni correction (*n *= 68 CpGs, *p *< 1.40 × 10^–7^). Blue crosses represent CpG sites that met genome-wide significance (FDR *q *< 0.05) and showed decreased methylation in infants whose mothers smoked. Red triangles represent CpG sites that met genome-wide significance and showed increased methylation in infants whose mothers smoked.

*Epigenome-wide association study*. Epigenome-wide association results based on model 1 are presented in Figure 1. In addition, a volcano plot and quantile–quantile (Q-Q) plot are provided as Supplemental Material (Figures S1–S2). Further adjustment for potential confounders in model 2 (maternal alcohol use, maternal education, maternal age at delivery, maternal folic acid supplement use, infant’s birth weight, and five different leukocyte cell type proportions) did not substantively affect the results as assessed by examining the correlation between the *p*-values and β-coefficients across all CpGs tested (see Supplemental Material, Figure S3). The more parsimonious model, model 1, was therefore selected as the primary model. Of 357,320 CpG sites tested for association with maternal smoking, we identified 185 CpG sites with altered methylation at genome-wide significance (FDR *q* < 0.05) (see Supplemental Material, Tables S2–S3) found within 110 gene regions. The term “gene region” was used to annotate CpGs located within or outside the transcribed region of a gene. The gene region for each CpG probe was defined as the nearest gene if the CpG site was intergenic or the gene itself if the CpG site was located within the gene body (see Supplemental Material, Table S3). Comparing exposed and unexposed infants, 43% of the 185 CpGs had decreased methylation (negative β-coefficient), and 57% had increased methylation (positive β-coefficient). The average percentage change in methylation (mean β_smokers_ – mean β_nonsmokers_) was small, ranging from –8% to 7% with a mean of –2% for sites with decreased methylation and 2% for sites showing increased methylation. Further annotation of the significant CpG sites, including distance to the closest transcription start site (TSS) and whether it is located within a CpG island, shelf, or shore is provided (see Supplemental Material, Table S4). We compared the distribution of the 185 differentially methylated CpG sites among genomic regions (CpG island, shelf, shore, or non-island), using the Illumina HumanMethylation450 BeadChip as a reference (see Supplemental Material, Table S5). In particular, we observed a significant enrichment for CpG shores when we examined all 185 CpGs (chi-square test, *p* = 0.002); the same thing was observed when the analysis was restricted to those 80 sites with decreased methylation (chi-square test, *p* = 0.003).

The relationship between the extent of smoke exposure and the level of methylation was also assessed. Of the 185 genome-wide significant CpG sites, only 107 CpGs (58%) showed evidence of a dose–response relationship (see Supplemental Material, Figure S4 and Table S6). When our results were restricted to the 23 novel CpG sites confirmed at the site level or gene level (Table 3), 18 of the sites (78%) showed a dose–response relationship. Expanding that list of 23 CpGs to include all 62 CpG sites confirmed at the site level or gene level, 49 (79%) met the criteria for a dose–response relationship. Further, CpGs with the smallest *p*-values had the highest proportion of dose-response relationships (see Supplemental Material, Table S7), with 91% of the CpGs showing a dose–response relationship with *p* < 1 × 10^–10^.

**Table 3:** Novel CpG sites confirmed at the site or gene level.^*a*^

Probe	Chromosome	Basepair (hg19)	Nearest gene	Model 1		Replication: 450K^*c*^ *p*-value
				Coefficient^*b*^	*p*-Value	
cg25189904	1	68299493	*GNG12*	–0.02	9.06 × 10^–9^*	4.96 × 10^–6^
cg26764244^*d*^	1	68299511	*GNG12*	–0.023	6.68 × 10^–10^*	4.47 × 10^–6^
cg18703066^*d*^	2	105363536	*LOC284998*	–0.006	7.63 × 10^–12^*	3.42 × 10^–7^
cg18096987	3	11623873	*VGLL4*	–0.018	4.10 × 10^–8^*	2.20 × 10^–6^
cg20344448^*d*^	10	14372431	*FRMD4A*	0.015	2.97 × 10^–9^*
cg11813497^*d*^	10	14372879	*FRMD4A*	0.024	6.85 × 10^–9^*	3.48 × 10^–6^
cg25464840^*d*^	10	14372910	*FRMD4A*	0.022	3.15 × 10^–15^*
cg15507334^*d*^	10	14372913	*FRMD4A*	0.02	2.43 × 10^–13^*
cg00029284^*d*^	12	111731203	*CUX2*	–0.009	9.62 × 10^–6^	1.53 × 10^–6^
cg08698721^*d*^	14	101294147	*MEG3*	0.013	1.66 × 10^–5^	2.92 × 10^–6^
cg04291079^*d*^	14	101294430	*MEG3*	0.013	1.67 × 10^–7^
cg00253658^*d*^	16	54210496	*FTO*	0.059	1.37 × 10^–17^*	9.64 × 10^–7^
cg26681628	16	54210550	*FTO*	0.023	4.05 × 10^–13^*
cg03687532	16	54228358	*FTO*	0.012	2.41 × 10^–5^
cg07339236^*d*^	20	50312490	*ATP9A*	–0.013	2.21 × 10^–18^*	1.38 × 10^–7^
cg16517298	1	230413174	*GALNT2*	–0.027	6.09 × 10^–6^
cg19727396^*d*^	1	230415185	*GALNT2*	–0.018	1.15 × 10^–5^
cg24591105^*d*^	1	230415225	*GALNT2*	–0.024	1.26 × 10^–7^*
cg00589617^*d*^	1	230415343	*GALNT2*	–0.029	1.07 × 10^–7^*
cg05697274^*d*^	1	230415377	*GALNT2*	–0.026	1.75 × 10^–8^*
cg24250902^*d*^	1	230415547	*GALNT2*	–0.032	1.13 × 10^–8^*
cg03144619^*d*^	1	230415668	*GALNT2*	–0.037	2.17 × 10^–9^*
cg09368188^*d*^	1	245330018	*KIF26B*	0.026	1.54 × 10^–7^
^***a***^Site/gene region was not part of the 26 sites/10 gene regions that met genome-wide significance in Joubert et al. 2012 (Bonferroni correction, *p *< 1.06 × 10^–7^) but was confirmed using their results containing 74 additional nominally significant sites/69 gene regions. ^***b***^A negative coefficient indicates reduced methylation in infants whose mothers smoked. ^***c***^Replication at the site level using a previously published study (Joubert et al. 2012) that used Illumina HumanMethylation450 BeadChips. Those sites without direct confirmation were included if they were genome-wide significant in our study and a different CpG site in the same gene region was identified by Joubert et al. (2012) (e.g., *GALNT2*). ^***d***^Met our criteria for dose response (see “Methods,” “Tobacco smoke exposure,” for details). *Met Bonferroni correction (*p *< 1.4 × 10^–7^).						

**Figure 2:**
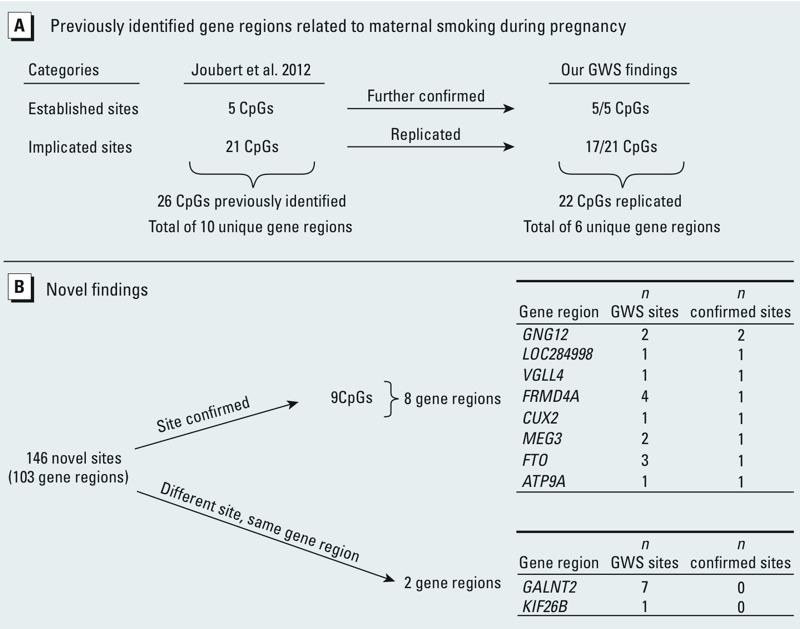
Replication of previously identified maternal smoking-related CpG sites (*A*) and confirmation of novel findings (*B*). GWS, genome-wide significant. (*A*) Twenty-six GWS CpG sites (Bonferroni correction: *p *< 1.06 × 10^–7^) related to maternal smoking during pregnancy were previously identified. Using our 185 GWS CpG sites (FDR *q *< 0.05) related to maternal smoking during pregnancy, we further confirmed all 5 CpGs that had been previously replicated using a separate study population (Bonferroni correction for 26 tests; *p *< 0.0019) as described by Joubert et al. (2012). Further, we replicated 17 of the 21 CpGs that were not directly replicated in their small replication population using a Bonferroni correction for 26 tests. In total, 22 CpGs (6 unique gene regions) of the 26 CpGs (10 unique gene regions) were either further confirmed or replicated at the site level. An additional 17 CpGs, located in 7 of the 10 previously implicated or established gene regions, met genome-wide significance in our study, bringing the total to 39 CpGs in 7 gene regions: 22 CpGs replicated at the site level and 17 CpGs replicated at the gene level. (*B*) Novel findings included those CpG sites in gene regions not previously identified as shown in (*A*). Publicly available results (Joubert et al. 2012) were used to confirm our findings. We considered two scenarios: The exact CpG was confirmed versus the exact CpG was not confirmed, but other CpGs were identified in the same gene region.

*Replication using publically available EWAS results*. We identified 185 CpGs (corresponding to 110 gene regions) with altered methylation at genome-wide significance (FDR *q* < 0.05; see Supplemental Material, Tables S2–S3). To replicate our findings, we used results from the only previous maternal smoking study using Illumina HumanMethylation450 BeadChips [results obtained from Supplemental Table S1 in Joubert et al. (2012); see also Supplemental Material, Table S8]. In the study by Joubert et al. (2012), which used a conservative Bonferroni correction for 473,844 tests (*p* < 1.06 × 10^–7^) as the criterion for genome-wide significance, 26 CpGs were reported as genome-wide significant. We first describe our confirmation of those 26 CpGs (10 gene regions) that we further classify as either established (*n* = 5; identified previously at genome-wide significance and replicated using an independent population) or implicated (*n* = 21; identified previously at genome-wide significance, currently lacking replication). We confirmed the 5 of 26 CpG sites (3 gene regions: *AHRR*, *CYP1A1*, and *GFI1*) that were additionally replicated (Bonferroni correction for 26 tests: *p* < 0.0019) by Joubert and colleagues using an independent population of infants born to 18 smoking and 18 nonsmoking mothers from the Newborn Epigenetics Study (NEST) (Figure 2A) (Joubert et al. 2012). In addition, we replicated 17 of the 21 CpGs that were previously identified at genome-wide significance, but not directly replicated using 36 mothers from NEST (*p* < 0.0019) although these had replication *p*-values ranging from 0.002 to 0.066 (Figure 2A). Replication of those 17 sites established an additional 3 gene regions (*MYO1G*, *CNTNAP2*, *LOC100507468*) related to maternal smoking during pregnancy. In addition to the 22 CpGs replicated as described above, an additional 17 CpGs, located in 7 of the 10 previously implicated or established gene regions met genome-wide significance in our study, bringing the total number to 39 CpGs in 7 gene regions that were confirmed at either site level (22 CpGs with exact CpG site confirmation; Figure 2A) or gene level (17 CpGs without exact CpG site confirmation but in or near one of the 10 genes). In addition to the 26 maternal smoking-related CpGs previously reported, Joubert et al. (2012) provided results for 74 nominally significant CpGs (part of their top 100 findings) representing 69 gene regions that did not meet their strict criteria for genome-wide significance (Bonferroni correction, *p* < 1.06 × 10^–7^). We used this set of results to serve as an independent replication set for our novel findings [146 CpGs corresponding to 103 gene regions not previously implicated (Figure 2B; also see Supplemental Material, Table S8)], which we describe in detail below.

Using the publically available results consisting of 74 nominally significant CpGs, we find confirmation of 9 CpGs in 8 gene regions (*GNG12*, *LOC284998*, *VGLL4*, *FRMD4A*, *CUX2*, *MEG3*, *FTO*, and *ATP9A*). Further, we identified genome-wide significant CpGs in 2 additional gene regions (*GALNT2* and *KIF26B*) where a different CpG site in the same gene region showed altered DNA methylation (i.e., the exact CpG sites were not identical). In total, we identified 8 CpGs in these 2 gene regions and 15 CpGs in the 8 gene regions that met genome-wide significance in our study (FDR *q* < 0.05, Figure 2B, Table 3). This set of 10 genes is the focus of the discussion below. Five of these 10 gene regions—*GNG12*, *GALNT2*, *FRMD4A*, *MEG3*, and *FTO*—contain multiple differentially methylated CpG sites at genome-wide significance. Notably, all confirmed sites showed the same direction of effect in both studies. The remaining 123 CpGs (representing 93 gene regions) that achieved genome-wide significance (FDR *q* < 0.05) in our study require further confirmation.

*Smoking-related methylation changes in adults and newborns*. We also considered our results in relation to three published studies of adult smoking that used the Illumina HumanMethylation450 BeadChip for their primary analysis (Monick et al. 2012; Shenker et al. 2012; Zeilinger et al. 2013). The CpG sites related to adult smoking include: *a*) 187 genome-wide significant sites [*p* ≤ 1 × 10^–7^; obtained from Zeilinger et al. (2013), their Table S2]; *b*) top findings for breast (*n*_sites_ = 17) and colon cancer (*n*_sites_ = 19) EWASs of adult smoking [*p* < 1 × 10^–5^; obtained from Shenker et al. (2012), their Table S3], and *c*) top 30 differentially methylated CpGs identified in lymphoblasts and alveolar macrophages [some, but not all are genome-wide significant; obtained from Monick et al. (2012), their Tables 2 and 5]. Using these lists, we examined the overlap between smoking-related DNA methylation in adults and newborns. Of the 185 genome-wide significant CpG sites identified in our study, 21 CpGs (6 gene regions) were identified with the same direction of effect in at least one of the three adult smoking studies (see Supplemental Material, Table S8).

We next sought to more generally compare the established smoking-related genes in adults and newborns (see Supplemental Material, Table S8). Ten gene regions were identified as among the top findings in at least two of the three adult studies. We compared the two newborn studies using gene regions from the 185 CpGs in our study and the 100 CpGs reported by Joubert et al. (2012) and found 17 gene regions in common. In both the adult and newborn comparisons, the same gene region was implicated, but the exact CpG site may differ between studies. Of the 10 established gene regions identified in multiple adult studies, 4 were identified in both newborn studies (*AHRR*, *GNG12*, *GFI1*, *CNTNAP2*). The remaining six gene regions (*LOC100131546*, *TMEM51*, *IER3*, *GNA12*, *LRP5*, *F2RL3*) were restricted to adults only. In contrast, of 17 established gene regions identified in both newborn studies, 6 were reported in at least one adult study (*AHRR*, *CNTNAP2*, *GNG12*, *GFI1*, *ATP9A*, *MYO1G*), whereas 11 (*LOC284998*, *FTO*, *CUX2*, *CYP1A1*, *VGLL4*, *KIF26B*, *MEG3*, *FRMD4A*, *GALNT2*, *LOC100507468*, *EXT1*) were restricted to newborns only.

Our final comparison between adult and newborn findings was related to the direction of methylation change. In contrast to newborns, where 43% of CpGs had decreased methylation, > 70% of the CpGs identified in adult smokers had decreased methylation. However, there is some limited evidence, at least in adults, that this finding may be tissue specific (Monick et al. 2012).

## Discussion

We conducted an EWAS investigating alterations in DNA methylation in infants indirectly exposed *in utero* to maternal tobacco smoke. We identified 185 CpG sites that met genome-wide significance (FDR *q* < 0.05). Publicly available results from a recent EWAS of maternal smoking were used to confirm some of our findings (Joubert et al. 2012). We also described similarities and differences between smoking-related methylation changes in adults and newborns.

Our study confirmed 5 of 5 previously replicated maternal smoking-related CpG sites representing 3 gene regions. We also replicated another 17 of 21 CpGs, representing 3 additional gene regions, that had achieved genome-wide significance in the previous study but were not replicated in their small replication population using a Bonferroni correction for multiple tests (Joubert et al. 2012). Most of these CpGs, however, had *p*-values < 0.05. Most important, we provide new findings of 8 gene regions associated with maternal smoking where, using the publicly available results from Joubert et al. (2012), there was exact CpG site confirmation and 2 gene regions where different CpGs showed evidence of association (Table 3). From this set of 10 genes with newly established links to maternal smoking, the most noteworthy include *MEG3* (maternally expressed 3), *FRMD4A* (FERM domain containing 4A), *ATP9A* (ATPase, class II, type 9A), and *GALNT2* (UDP-*N*-acetyl-alpha-d-galactosamine:polypeptide *N*-acetylgalactosaminyltransferase 2), discussed in detail below.

In *MEG3* we find 2 genome-wide significant CpGs and 1 nominally associated CpG with increased methylation in infants of mothers who smoked (see Supplemental Material, Figure S5). *MEG3* is an imprinted gene (paternal allele silenced, maternal allele expressed) that encodes a long noncoding RNA implicated in tumorigenesis (Benetatos et al. 2011) and embryonic development (Zhou et al. 2010). Previous studies suggest a potential link between *MEG3*, tobacco smoke, and development. Expression of *MEG3* is down-regulated in immortalized human bronchial epithelial cells in response to treatment with cigarette smoke condensate but can be reactivated following 5-aza-2-deoxycytidine treatment (Hu et al. 2009). This suggests that the smoking-related decrease in expression may be associated with increased DNA methylation, consistent with the direction of effect found in newborns of mothers who smoke. Related to development, maternal cigarette smoking during pregnancy causes reduced birth weight (Suter et al. 2013), and expression of *MEG3* is down-regulated in placentae with intrauterine growth restriction (McMinn et al. 2006), as was observed in human bronchial epithelial cells treated with cigarette smoke condensate. However, unlike the direction of effect observed in the previous study, *MEG3* is up-regulated in placentas of women smokers compared with nonsmokers (Votavova et al. 2011). Given these inconsistent findings, additional studies are needed to clarify this relationship.

The *DLK1*/*MEG3* imprinting domain on 14q32.2 has two differentially methylated regions (DMRs), the MEG3-DMR (secondary, postfertilization-derived DMR; located near our smoking-associated CpGs) and IG-DMR (primary, germline-derived intergenic DMR) (Skaar et al. 2012) that are hypothesized to act as imprinting control centers in the body and placenta, respectively (Kagami et al. 2010). Although the mechanism by which the MEG3-DMR regulates imprinting is not well understood, CTCF (CCCTC-binding factor) is thought to be involved (Skaar et al. 2012), perhaps by modulating chromatin boundary formation (Wylie et al. 2000). The *MEG3* smoking-associated CpGs identified in our study are located at the border of the MEG3-DMR immediately adjacent to two putative CTCF binding sites (Wylie et al. 2000; also see Supplemental Material, Figure S5). If increased methylation in newborns of smokers inhibits CTCF binding, it could result in decreased expression of *MEG3* and other maternally expressed genes, as well as increased expression of paternally expressed genes in that region (Kagami et al. 2010). Dysregulation of MEG3 could potentially affect embryonic development and, if the altered methylation state persists for an extended period of time, have long-term health effects.

We identified altered methylation of CpGs near two genes, *FRMD4A* and *ATP9A*, associated with nicotine (Yoon et al. 2012) and substance dependence (Johnson et al. 2008), and the ability to quit smoking (Uhl et al. 2008). Increased methylation at 4 CpGs in newborns of smokers was observed in *FRMD4A*, a scaffolding protein implicated in the regulation of epithelial polarity (Ikenouchi and Umeda 2010). Decreased methylation with smoking exposure was found at 1 CpG in *ATP9A*, a class 2 P4-ATPase that localizes to endosomes and the *trans*-Golgi network (Takatsu et al. 2011). Although findings are not consistent across all studies, maternal smoking during pregnancy has been previously associated with smoking-related traits in offspring (e.g., nicotine dependence) even after accounting for postnatal exposure to tobacco smoke (Al Mamun et al. 2006; Kandel et al. 1994; Kardia et al. 2003; Lieb et al. 2003; Selya et al. 2012); however, the possibility cannot be ruled out that these associations are attributable to unmeasured confounding. An animal study provides further support for an association by showing a neurotoxicant effect of prenatal nicotine exposure in rats that persisted into adolescence (Abreu-Villaca et al. 2004). Authors suggested that if similar, persistent alterations occurred in offspring of mothers who smoked during pregnancy, this could contribute to an increased susceptibility of nicotine dependence in adolescence. Thus, if the altered methylation states of *FRMD4A* and/or *ATP9A* persisted through adolescence, these could potentially contribute to the observed relationship between maternal smoking and smoking-related traits, such as nicotine dependence, in offspring.

In *GALNT2* we identified 7 CpGs at genome-wide significance that showed decreased methylation in newborns of smokers. *GALNT2* is associated with lipid levels (Teslovich et al. 2010), insulin signaling (Marucci et al. 2013a), hyperglycemia (Marucci et al. 2013b), and the malignant behavior of hepatocellular carcinoma (Wu et al. 2011). *GALNT2* may also play a role in extravillus trophoblast invasion, which is important for placental development (Liao et al. 2012). Aberrant DNA methylation of *GALNT2*, which has so far been identified only in newborns exposed and not adults, may affect the development of the placenta and could subsequently influence the health of the fetus.

In this study we described some unique and common aspects of smoking-related methylation changes in adults and newborns. Less than half of the maternal smoking–related genes that are now well established in newborn studies were identified in any of the three published adult smoking studies. Moreover, some of the best-established adult smoking–related gene regions (e.g., *F2RL3*) were not among the top results from either of the newborn studies. These observations suggest that there could be distinct effects of direct tobacco smoke exposure in adults versus indirect tobacco smoke exposure *in utero*. There are several plausible explanations for this observation:

Age-related susceptibility: The epigenome of the developing embryo and fetus is thought to be particularly susceptible to environmental exposures because many epigenetic changes are occurring along with rapid cell division (Perera and Herbstman 2011).
Tissue-specific alterations related to age at exposure: Exposure during development may be more likely to induce widespread changes (i.e., many tissues affected), whereas exposure during adulthood may be more likely to induce restricted changes, perhaps identifiable in only one or a few tissues (Rakyan et al. 2011).
Exposure variability: The exposure itself could vary with respect to duration. In addition, the fetus is indirectly exposed to tobacco smoke, so the actual exposure is dependent on a number of factors, including the ability of the placenta and the mother to metabolize and detoxify components of the cigarette smoke.
Age-related differences in the response to exposure: Response to an environmental exposure, such as tobacco smoke, may vary by age due to differences in one’s metabolism and capacity to detoxify the components of tobacco smoke (Aldridge et al. 2003). For example, in response to tobacco smoke, age-dependent expression (Eke et al. 1997) of genes involved in metabolizing components of tobacco smoke has been reported.
Power of the studies: It is also possible that future newborn and adult studies with larger sample sizes will find more overlap of these genes.

There are several limitations to our study. We were able to confirm only some of our findings using publicly available results. Our confirmations were based on the top 100 sites described in a previously published study (Joubert et al. 2012), so we are unable to comment on the total number of CpGs that are currently lacking replication, given the fact that we did not have access to a complete replication set. Even with this limited list we were able to identify 10 new genes related to maternal smoking, including some with biologically plausible functions. Our correction for multiple testing was based on a reduced number of probes after filtering out the least variable 20% of CpGs. The alternative of filtering by degree of difference may induce FDR bias, but there is no evidence of such bias when filtering by variability (van Iterson et al. 2010). As a further check, we compared our results with and without filtering; the findings were essentially unchanged. Of the 23 novel CpG sites confirmed at the site or gene level (Table 3), all but one remained significant at a genome-wide level without filtering (FDR *q* < 0.05), whereas the one exception (cg03687532) had an FDR *q* = 0.06. These additional results provide some reassurance about the robustness of our conclusions. Another potential limitation is that we used self-reported smoking as opposed to a more objective measure, such as cotinine. However, self-reported smoking is generally considered to be a valid measure of smoking. Self-reported maternal smoking and plasma cotinine concentrations were compared in the Mother and Child Cohort Study, another population-based Norwegian study conducted during 2002–2003. That study reported a high level of validity for the self-reported data (Kvalvik et al. 2012). In our study, mean birth weight was reduced with maternal smoking (Table 2), consistent with the usual expected effect of smoking on the fetus. If there was underreporting of smoking during pregnancy, this type of misclassification would only reduce our power, and would not explain our detection of 185 CpG sites at genome-wide significance. Furthermore, in the absence of further validation (e.g., pyrosequencing) and functional work, it is not known whether these changes are biologically relevant or which gene or genes they affect, if any. It also remains to be seen whether these small changes in DNA methylation will be of clinical relevance.

## Conclusions

Consistent with previous reports, we found maternal smoking during pregnancy to be associated with an altered DNA methylation profile in infants. Of particular interest were findings in *FRMD4A*, *ATP9A*, *GALNT2*, and *MEG3*—genes that are implicated in processes related to nicotine dependence, smoking cessation, and placental and embryonic development. Further, we described some unique aspects of DNA methylation changes in adults compared with newborns. Direct and indirect *in utero* smoke exposure appear to elicit differential epigenetic responses that may be attributable to age-related differences in susceptibility to tobacco smoke, tissue-specific alterations related to age at exposure, variation in the exposure itself, and/or age-related differences in the response to exposure. It remains to be seen whether the small changes in DNA methylation identified (mean Δβ = ± 2%) are biologically or clinically relevant. Future studies examining the persistence of DNA methylation changes due to an *in utero* exposure throughout life, and particularly associations of these changes with health phenotypes later in life (i.e., DNA methylation as a mediator of health effects), will be of particular interest.

## Supplemental Material

(1.7 MB) PDF
