# Supplementary material for: Identification of DNA Methylation Changes in Newborns Related to Maternal Smoking during Pregnancy
Source: Environ Health Perspect. 2014 Jun 6;122(10):1147–53. doi: 10.1289/ehp.1307892 (PMC4181928; doi:10.1289/ehp.1307892)
Supplement: (1.7 MB) PDF [file ehp.1307892.s001.508.pdf]

Environmental Health Perspectives doi:10.1289/ehp.1307892

Supplemental Material

Identification of DNA Methylation Changes in Newborns Related to Maternal Smoking during Pregnancy

Christina A. Markunas, Zongli Xu, Sophia Harlid, Paul A. Wade, Rolv T. Lie, Jack A. Taylor, and Allen J. Wilcox

Table of Contents **Page**

Methods 3

Data generation 3

Data cleaning and quality assessment 3

Data pre-processing 4

Supplemental tables 6

Table S1. Newborn cell proportion estimates (mean ± SD) according to maternal smoking (non-active or active) 6

Table S2. Genome-wide significant CpG sites: comparison of Model1 and Model2 7

Table S3. CpG probe annotation 15

Table S4. CpG probe annotation 23

Table S5. Distribution of differentially methylated CpG sites by genomic region [n (%)] 31

Table S6. Genome-wide significant CpG sites: dose-response evaluation by maternal smoking status 32

Table S7. Percentage of CpGs showing a dose-response relationship 40

Table S8. Genome-wide significant CpG sites: replication using previously published studies 41

Table S9. Technical replicate assessment 47

Supplemental figures 48

Figure S1. Volcano plot 48

Figure S2. Quantile-quantile (Q-Q) 49
Figure S3. Comparison of results from Model1 and Model2 50
Figure S4. Genome-wide significant CpG sites that showed a dose-response relationship 51
Figure S5. MEG3 gene diagram 52
References 53

Methods

Data generation

Genomic DNA was extracted from whole blood using the automated Autopure LS system (Gentra, Minneapolis, MN). Extracted DNA was quantified using Quant-iT^{TM} PicoGreen dsDNA reagents (Invitrogen, Grand Island, NY) and stored at -20°C. One microgram of DNA was bisulfite converted using the EZ DNA Methylation kit (Zymo Research, Irvine, CA) following the manufacturer's protocol. In total, 898 newborn and 60 technical control samples (replicates and samples with varying levels of known DNA methylation) were run on Illumina HumanMethylation450 BeadChips (San Diego, CA) according to the manufacturer's instructions at the NIH Center for Inherited Disease Research. The Illumina HumanMethylation450 BeadChip contains 485,577 CpG sites which cover 99% of RefSeq genes and 96% of CpG islands.

Data cleaning and quality assessment

Samples were excluded if determined to be outliers based on visual inspection of a density plot using raw β-values (ratio of methylated to total signal), if less than 95% of CpG probes were detectable (Illumina detection p-value < 0.05), or if sex was ambiguous. In total, six samples were excluded due to poor performance based on Illumina's bisulfite conversion system controls (intensity value < 5500), one sample was excluded as a clear outlier in the raw β-value density plot, one sample was excluded due to ambiguous sex plus poor bisulfite conversion efficiency, and one sample was excluded as the mother was a snuff tobacco user. These exclusions left 889 samples for analysis ($N_{smokers}$= 287, $N_{non\text{-}smokers}$=602). We also conducted filtering of CpG probes. To avoid altered methylation measures due to SNPs, 20,869 CpG probes were excluded with SNPs present at their target site (Price et al. 2013). Furthermore, CpGs on sex chromosomes

were excluded and a non-specific filtering step was applied to exclude the least variable 20% of CpG probes as determined by the interquartile range of methylation β-values. After exclusion of these probes, a total of 357,320 CpGs remained for the association analysis. Additionally, to reduce the effect of outliers for each CpG probe, β-values that were more than 3 standard deviations from the mean were excluded. In addition, methylation levels that were deemed undetectable (Illumina detection p-value > 0.05) were excluded. Further data assessment was conducted by examining the correlation within the 20 duplicate sample pairs (Table S9).

Data pre-processing

Raw intensity data were obtained using the Illumina GenomeStudio methylation module (version 2011.1). At each CpG site on the array, methylation status was determined based on intensity measures corresponding to unmethylated (U) or methylated (M) signal. The Illumina HumanMethylation450 BeadChip contains two probe types: Infinium Type I (2 probe types, 1 color channel) and II (1 probe type, 2 color channels). As the Type II probes use two color channels to assess methylation, dye bias was corrected using the normalization function (normalizeMethyLumiSet), provided in the R package, methylumi (Davis et al. 2013). Before association analysis, the M and U intensity values for Type I and II probes were separately background adjusted (4 separate groups) using the robust multi-array average (RMA) method (Irizarry et al. 2003) and quantile normalized using the normalization function (normalize.quantiles), provided in the R package, Affy (Bolstad et al. 2003). The β-value (M/(M+U+100)) was then computed and used in the association analysis. Prior to running the robust linear regression model, residuals were calculated for each CpG probe from linear regression models adjusting for infant's sex, and two technical factors: batch (96-well plate) and bisulfite efficiency. Missing β-values generated due to the exclusion of outliers (see above for

description) were first imputed using the KNN method (10 nearest markers) as implemented in the R package, impute (Hastie et al. 2014). Following adjustment of the data for technical factors and sex, outliers were re-set to missing and residuals were used as input for the primary analysis. Additional covariates thought to be potential confounders (e.g. facial cleft status) were included in the robust linear regression model, along with maternal smoking status, as described in the methods section (Analysis) of the main manuscript.

Supplemental tables

Table S1. Newborn cell proportion estimates (mean ± SD) according to maternal smoking (non-active or active)

**Cell subtype**[a]	**Non-active**[b]	**Active**[b]	**P-value**[c]
T-cells	0.49 ± 0.11	0.49 ± 0.11	0.36
Granulocytes	0.31 ± 0.12	0.31 ± 0.11	0.48
Monocytes	0.10 ± 0.06	0.09 ± 0.06	0.73
B cells	0.09 ± 0.04	0.09 ± 0.04	0.70
NK cells	0.02 ± 0.04	0.02 ± 0.05	0.83

[a]5 different cell type proportions were estimated using dataset, GSE35069. [b]Cell proportion estimates were normalized to equal 1.

[c]Based on a linear mixed model adjusted for sex and cleft status as fixed effects and BeadChip as a random effect.

Table S2. Genome-wide significant CpG sites: comparison of Model1 and Model2.[a]

Probe	Model1[b] Coeff	Model1[b] *P*-value	Model1[b] *q*-value	Model2[c] Coeff	Model2[c] *P*-value	Model2[c] *q*-value	Δ β-value[d]
cg05575921	-0.0460	1.45E-69*	5.17E-64	-0.0466	6.67E-65*	2.38E-59	-0.043
cg04180046	0.0510	1.53E-40*	2.73E-35	0.0500	5.08E-38*	9.07E-33	0.052
cg09935388	-0.0860	2.39E-34*	2.85E-29	-0.0848	9.36E-32*	1.11E-26	-0.084
cg25949550	-0.0200	1.39E-31*	1.24E-26	-0.0198	7.15E-31*	6.39E-26	-0.019
cg12876356	-0.0690	2.22E-31*	1.59E-26	-0.0699	4.49E-30*	3.21E-25	-0.077
cg12803068	0.0530	3.58E-31*	2.13E-26	0.0525	1.22E-29*	7.28E-25	0.053
cg18146737	-0.0650	3.83E-30*	1.96E-25	-0.0657	5.46E-29*	2.79E-24	-0.075
cg18316974	-0.0330	3.47E-29*	1.55E-24	-0.0339	3.72E-27*	1.66E-22	-0.044
cg21161138	-0.0190	5.51E-26*	2.19E-21	-0.0181	2.00E-21*	7.14E-17	-0.02
cg19089201	0.0170	1.62E-23*	5.79E-19	0.0163	2.22E-21*	7.21E-17	0.02
cg14179389	-0.0680	3.38E-23*	1.10E-18	-0.0664	2.75E-21*	8.18E-17	-0.065
cg06338710	-0.0230	1.96E-21*	5.83E-17	-0.0232	9.38E-22*	3.72E-17	-0.031
cg22937882	0.0130	2.76E-21*	7.58E-17	0.0139	7.93E-21*	2.18E-16	0.013
cg09662411	-0.0340	1.06E-19*	2.69E-15	-0.0350	3.77E-20*	9.63E-16	-0.043
cg22132788	0.0150	2.11E-19*	5.04E-15	0.0147	6.92E-18*	1.37E-13	0.016
cg07339236	-0.0130	2.21E-18*	4.94E-14	-0.0138	7.28E-19*	1.63E-14	-0.013
cg05549655	0.0320	7.92E-18*	1.66E-13	0.0312	6.58E-16*	1.12E-11	0.036
cg11924019	0.0290	9.40E-18*	1.87E-13	0.0286	6.99E-16*	1.13E-11	0.033
cg00253658	0.0590	1.37E-17*	2.57E-13	0.0580	8.60E-17*	1.62E-12	0.063
cg22549041	0.0590	5.94E-17*	1.06E-12	0.0581	9.64E-16*	1.50E-11	0.066
cg08606254	0.0200	2.14E-15*	3.65E-11	0.0215	2.36E-16*	4.22E-12	0.021

Probe	Model1[b] Coeff	Model1[b] *P*-value	Model1[b] *q*-value	Model2[c] Coeff	Model2[c] *P*-value	Model2[c] *q*-value	Δ β-value[d]
cg25464840	0.0220	3.15E-15*	5.11E-11	0.0262	8.03E-20*	1.91E-15	0.023
cg12101586	0.0390	5.38E-15*	8.36E-11	0.0393	1.74E-13*	2.49E-09	0.043
cg15507334	0.0200	2.43E-13*	3.62E-09	0.0238	5.78E-18*	1.22E-13	0.022
cg26681628	0.0230	4.05E-13*	5.79E-09	0.0224	2.13E-13*	2.93E-09	0.023
cg18092474	0.0420	5.47E-13*	7.51E-09	0.0425	4.29E-12*	5.11E-08	0.05
cg23067299	0.0220	8.94E-13*	1.18E-08	0.0249	2.92E-14*	4.35E-10	0.022
cg13570656	0.0420	9.63E-13*	1.23E-08	0.0417	1.99E-11*	2.04E-07	0.044
cg11429111	0.0310	1.04E-12*	1.29E-08	0.0315	7.32E-12*	8.18E-08	0.034
cg01952185	0.0290	2.55E-12*	3.04E-08	0.0309	2.59E-12*	3.30E-08	0.031
cg18703066	-0.0060	7.63E-12*	8.79E-08	-0.0059	1.02E-09*	9.09E-06	-0.004
cg11902777	-0.0080	1.13E-11*	1.26E-07	-0.0087	9.33E-12*	1.01E-07	-0.009
cg18132363	-0.0270	8.92E-11*	9.53E-07	-0.0272	3.94E-10*	3.70E-06	-0.029
cg15325070	0.0180	9.07E-11*	9.53E-07	0.0188	1.71E-10*	1.70E-06	0.018
cg10399789	-0.0160	5.74E-10*	5.66E-06	-0.0177	2.72E-12*	3.35E-08	-0.02
cg14817490	-0.0230	5.75E-10*	5.66E-06	-0.0248	1.07E-12*	1.42E-08	-0.021
cg01031101	0.0230	5.86E-10*	5.66E-06	0.0237	8.02E-10*	7.34E-06	0.026
cg26764244	-0.0230	6.68E-10*	6.28E-06	-0.0233	3.29E-10*	3.18E-06	-0.023
cg00213123	0.0120	7.47E-10*	6.85E-06	0.0116	1.24E-08*	8.87E-05	0.014
cg03796381	0.0150	1.17E-09*	1.05E-05	0.0136	2.11E-07	1.13E-03	0.015
cg10520740	-0.0090	1.86E-09*	1.62E-05	-0.0094	1.92E-09*	1.64E-05	-0.01
cg03144619	-0.0370	2.17E-09*	1.85E-05	-0.0356	4.24E-08*	2.86E-04	-0.038
cg20344448	0.0150	2.97E-09*	2.47E-05	0.0175	5.32E-12*	6.13E-08	0.015

Probe	Model1[b] Coeff	Model1[b] *P*-value	Model1[b] *q*-value	Model2[c] Coeff	Model2[c] *P*-value	Model2[c] *q*-value	Δ β-value[d]
cg01359532	0.0140	3.11E-09*	2.53E-05	0.0149	7.08E-09*	5.49E-05	0.015
cg10253847	0.0130	6.51E-09*	5.17E-05	0.0132	3.85E-08*	2.65E-04	0.015
cg11813497	0.0240	6.85E-09*	5.32E-05	0.0277	1.94E-11*	2.04E-07	0.029
cg25189904	-0.0200	9.06E-09*	6.89E-05	-0.0208	7.84E-09*	5.72E-05	-0.018
cg24250902	-0.0320	1.13E-08*	8.43E-05	-0.0322	4.70E-08*	3.00E-04	-0.031
cg13822849	0.0150	1.18E-08*	8.58E-05	0.0161	1.24E-09*	1.08E-05	0.015
cg23916896	-0.0220	1.24E-08*	8.87E-05	-0.0212	1.17E-07*	6.88E-04	-0.022
cg13834112	0.0230	1.46E-08*	1.00E-04	0.0247	4.34E-09*	3.52E-05	0.029
cg26889659	-0.0550	1.46E-08*	1.00E-04	-0.0506	4.86E-07	2.35E-03	-0.047
cg12806681	-0.0090	1.59E-08*	1.07E-04	-0.0084	4.28E-07	2.13E-03	-0.008
cg05697274	-0.0260	1.75E-08*	1.16E-04	-0.0253	1.16E-07*	6.88E-04	-0.024
cg18096987	-0.0180	4.10E-08*	2.67E-04	-0.0173	7.22E-09*	5.49E-05	-0.013
cg10312186	-0.0260	4.40E-08*	2.81E-04	-0.0245	5.96E-07	2.66E-03	-0.025
cg08097581	-0.0110	5.15E-08*	3.21E-04	-0.0110	7.52E-08*	4.63E-04	-0.012
cg07104557	-0.0140	5.21E-08*	3.21E-04	-0.0144	4.57E-08*	2.97E-04	-0.017
cg15578140	0.0140	5.56E-08*	3.32E-04	0.0150	7.57E-09*	5.64E-05	0.015
cg23335299	0.0250	5.58E-08*	3.32E-04	0.0269	5.90E-09*	4.68E-05	0.03
cg20244340	-0.0170	6.33E-08*	3.71E-04	-0.0156	1.71E-06	5.94E-03	-0.015
cg04459939	0.0240	6.91E-08*	3.98E-04	0.0278	2.50E-09*	2.08E-05	0.029
cg18694169	0.0190	9.62E-08*	5.46E-04	0.0188	4.79E-07	2.34E-03	0.02
cg00589617	-0.0290	1.07E-07*	5.89E-04	-0.0279	1.38E-06	5.18E-03	-0.029
cg04598670	-0.0210	1.07E-07*	5.89E-04	-0.0183	1.23E-05	2.72E-02	-0.016

Probe	Model1[b] Coeff	Model1[b] *P*-value	Model1[b] *q*-value	Model2[c] Coeff	Model2[c] *P*-value	Model2[c] *q*-value	Δ β-value[d]
cg24591105	-0.0240	1.26E-07*	6.83E-04	-0.0227	1.80E-06	6.14E-03	-0.026
cg04098479	0.0040	1.29E-07*	6.89E-04	0.0033	1.75E-06	6.01E-03	0.003
cg03453449	0.0180	1.31E-07*	6.89E-04	0.0191	5.21E-08*	3.26E-04	0.017
cg09368188	0.0260	1.54E-07	7.95E-04	0.0274	1.30E-07*	7.48E-04	0.03
cg04291079	0.0130	1.67E-07	8.53E-04	0.0139	1.41E-07	7.96E-04	0.013
cg05568941	0.0150	1.78E-07	8.94E-04	0.0175	1.60E-08*	1.12E-04	0.019
cg04478905	-0.0160	1.96E-07	9.70E-04	-0.0131	2.86E-05	4.77E-02	-0.013
cg15522425	0.0140	2.36E-07	1.16E-03	0.0150	4.45E-08*	2.94E-04	0.014
cg18621256	-0.0140	3.95E-07	1.91E-03	-0.0132	5.78E-06	1.56E-02	-0.015
cg26954197	0.0070	4.80E-07	2.29E-03	0.0072	5.28E-07	2.48E-03	0.006
cg03991871	-0.0090	5.01E-07	2.36E-03	-0.0090	5.06E-06	1.45E-02	-0.008
cg19631472	-0.0120	5.28E-07	2.45E-03	-0.0119	1.01E-06	4.04E-03	-0.011
cg16459265	-0.0100	6.22E-07	2.85E-03	-0.0081	4.53E-05	6.38E-02	-0.011
cg26119806	-0.0170	6.56E-07	2.97E-03	-0.0149	4.77E-05	6.50E-02	-0.017
cg03177666	0.0170	7.08E-07	3.16E-03	0.0172	1.45E-06	5.35E-03	0.019
cg17852385	0.0230	7.21E-07	3.18E-03	0.0228	2.75E-06	8.92E-03	0.026
cg25632883	-0.0360	7.61E-07	3.31E-03	-0.0405	1.01E-07*	6.09E-04	-0.035
cg20643029	-0.0130	7.70E-07	3.31E-03	-0.0111	3.39E-05	5.28E-02	-0.013
cg00794911	-0.0100	8.10E-07	3.45E-03	-0.0092	2.54E-05	4.44E-02	-0.011
cg02892925	0.0200	8.48E-07	3.56E-03	0.0189	1.78E-05	3.59E-02	0.018
cg09523275	0.0180	8.79E-07	3.65E-03	0.0175	3.47E-06	1.06E-02	0.022
cg17924476	0.0170	9.38E-07	3.85E-03	0.0192	1.43E-07	7.96E-04	0.02
cg15836046	0.0060	1.03E-06	4.19E-03	0.0064	5.86E-07	2.65E-03	0.005
cg12426652	-0.0090	1.18E-06	4.73E-03	-0.0077	6.04E-05	7.40E-02	-0.007
cg26516004	0.0080	1.20E-06	4.77E-03	0.0083	1.29E-06	4.90E-03	0.009

**Probe**	**Model1[b] Coeff**	**Model1[b] *P*-value**	**Model1[b] *q*-value**	**Model2[c] Coeff**	**Model2[c] *P*-value**	**Model2[c] *q*-value**	**Δ β-value[d]**
cg08626436	0.0320	1.57E-06	6.15E-03	0.0347	9.02E-07	3.70E-03	0.038
cg18753646	-0.0220	1.60E-06	6.22E-03	-0.0211	6.27E-06	1.65E-02	-0.019
cg07211044	0.0230	1.65E-06	6.32E-03	0.0212	3.03E-05	4.91E-02	0.025
cg23664708	-0.0170	1.70E-06	6.35E-03	-0.0165	3.11E-06	9.83E-03	-0.013
cg09743950	-0.0160	1.71E-06	6.35E-03	-0.0127	2.25E-04	1.55E-01	-0.016
cg18675097	0.0280	1.71E-06	6.35E-03	0.0277	3.15E-06	9.83E-03	0.032
cg26652413	0.0110	1.94E-06	7.16E-03	0.0119	4.02E-07	2.02E-03	0.013
cg18722086	0.0060	2.40E-06	8.68E-03	0.0047	8.36E-05	8.89E-02	0.005
cg01825213	0.0200	2.41E-06	8.68E-03	0.0222	9.27E-07	3.77E-03	0.019
cg25383593	0.0120	2.56E-06	9.09E-03	0.0113	1.51E-05	3.20E-02	0.013
cg20816361	0.0160	2.60E-06	9.09E-03	0.0175	1.24E-06	4.77E-03	0.015
cg10078511	0.0130	2.61E-06	9.09E-03	0.0140	1.57E-06	5.61E-03	0.013
cg06813382	0.0180	2.62E-06	9.09E-03	0.0187	2.56E-06	8.38E-03	0.019
cg06415891	0.0190	2.74E-06	9.41E-03	0.0208	1.14E-06	4.42E-03	0.018
cg17442776	0.0090	3.14E-06	1.06E-02	0.0104	8.25E-07	3.43E-03	0.008
cg23849826	0.0090	3.16E-06	1.06E-02	0.0103	6.28E-07	2.73E-03	0.01
cg08026735	0.0120	3.16E-06	1.06E-02	0.0117	8.56E-06	2.07E-02	0.012
cg11625488	-0.0220	3.70E-06	1.22E-02	-0.0229	8.24E-06	2.05E-02	-0.019
cg10841124	0.0040	3.79E-06	1.24E-02	0.0043	5.67E-06	1.55E-02	0.005
cg12937151	-0.0280	3.89E-06	1.26E-02	-0.0259	4.46E-05	6.38E-02	-0.026
cg16547579	-0.0160	3.94E-06	1.27E-02	-0.0124	7.35E-05	8.36E-02	-0.012
cg11096905	-0.0160	4.15E-06	1.32E-02	-0.0142	4.86E-05	6.53E-02	-0.015
cg24698536	-0.0260	4.22E-06	1.34E-02	-0.0257	1.16E-05	2.59E-02	-0.022
cg24239148	0.0070	4.69E-06	1.47E-02	0.0072	1.40E-05	2.99E-02	0.007
cg10673740	0.0190	5.08E-06	1.58E-02	0.0171	5.98E-05	7.37E-02	0.021
cg22057874	0.0130	5.25E-06	1.62E-02	0.0103	4.13E-05	6.07E-02	0.013
cg04640109	0.0110	5.43E-06	1.66E-02	0.0116	1.55E-06	5.60E-03	0.013

Probe	Model1[b] Coeff	Model1[b] *P*-value	Model1[b] *q*-value	Model2[c] Coeff	Model2[c] *P*-value	Model2[c] *q*-value	Δ β-value[d]
cg12492906	-0.0200	5.87E-06	1.78E-02	-0.0199	8.29E-06	2.05E-02	-0.02
cg11641006	0.0160	5.92E-06	1.78E-02	0.0172	3.39E-07	1.76E-03	0.014
cg11868728	0.0090	6.03E-06	1.80E-02	0.0094	4.27E-06	1.25E-02	0.007
cg16517298	-0.0270	6.09E-06	1.80E-02	-0.0261	7.86E-07	3.30E-03	-0.027
cg05593775	0.0160	6.18E-06	1.81E-02	0.0171	9.34E-06	2.20E-02	0.022
cg03354508	0.0110	6.85E-06	1.98E-02	0.0116	5.46E-06	1.54E-02	0.01
cg24173182	0.0220	6.86E-06	1.98E-02	0.0192	1.10E-04	1.07E-01	0.024
cg13784312	-0.0160	7.22E-06	2.06E-02	-0.0184	1.66E-06	5.82E-03	-0.02
cg26554302	0.0140	7.27E-06	2.06E-02	0.0126	6.99E-05	8.06E-02	0.02
cg22043296	-0.0120	7.42E-06	2.09E-02	-0.0107	1.28E-05	2.82E-02	-0.016
cg04800195	0.0030	7.56E-06	2.11E-02	0.0030	1.12E-04	1.09E-01	0.003
cg07249149	-0.0130	7.62E-06	2.11E-02	-0.0141	5.61E-06	1.55E-02	-0.015
cg15963463	-0.0110	7.69E-06	2.11E-02	-0.0101	5.95E-05	7.36E-02	-0.008
cg14086013	0.0120	7.74E-06	2.11E-02	0.0141	1.13E-06	4.42E-03	0.015
cg02010738	-0.0200	8.13E-06	2.20E-02	-0.0221	5.76E-07	2.64E-03	-0.019
cg18794473	0.0040	8.61E-06	2.30E-02	0.0039	6.40E-05	7.68E-02	0.003
cg26804595	-0.0100	8.61E-06	2.30E-02	-0.0102	8.51E-06	2.07E-02	-0.008
cg14027333	-0.0050	8.76E-06	2.32E-02	-0.0047	2.78E-05	4.69E-02	-0.004
cg02356218	-0.0140	9.03E-06	2.37E-02	-0.0126	6.51E-05	7.75E-02	-0.013
cg14349977	-0.0220	9.09E-06	2.37E-02	-0.0209	7.78E-05	8.66E-02	-0.021
cg09063262	-0.0220	9.37E-06	2.41E-02	-0.0198	1.83E-04	1.37E-01	-0.021
cg26441486	-0.0180	9.38E-06	2.41E-02	-0.0161	6.49E-05	7.75E-02	-0.019
cg00029284	-0.0090	9.62E-06	2.46E-02	-0.0081	1.01E-04	1.00E-01	-0.009
cg12160087	-0.0100	1.14E-05	2.88E-02	-0.0088	1.64E-04	1.29E-01	-0.01
cg19727396	-0.0180	1.15E-05	2.88E-02	-0.0172	3.58E-05	5.53E-02	-0.02
cg06949812	0.0130	1.20E-05	3.00E-02	0.0154	6.20E-07	2.73E-03	0.015
cg26103179	-0.0150	1.32E-05	3.27E-02	-0.0147	3.96E-05	5.87E-02	-0.012

Probe	Model1[b] Coeff	Model1[b] *P*-value	Model1[b] *q*-value	Model2[c] Coeff	Model2[c] *P*-value	Model2[c] *q*-value	Δ β-value[d]
cg09488203	0.0090	1.33E-05	3.27E-02	0.0099	2.37E-06	7.83E-03	0.009
cg13928411	0.0090	1.35E-05	3.29E-02	0.0098	3.22E-06	9.91E-03	0.008
cg16231923	0.0200	1.36E-05	3.30E-02	0.0200	2.58E-05	4.48E-02	0.023
cg20274009	0.0070	1.37E-05	3.32E-02	0.0073	5.67E-06	1.55E-02	0.004
cg22877366	-0.0200	1.40E-05	3.35E-02	-0.0175	1.71E-04	1.32E-01	-0.019
cg16449012	0.0110	1.42E-05	3.39E-02	0.0133	3.72E-07	1.90E-03	0.011
cg00433296	0.0100	1.43E-05	3.39E-02	0.0117	1.59E-06	5.64E-03	0.01
cg05656688	-0.0140	1.44E-05	3.40E-02	-0.0134	1.48E-05	3.15E-02	-0.015
cg08142858	0.0080	1.49E-05	3.49E-02	0.0092	1.47E-06	5.35E-03	0.005
cg25415695	0.0120	1.61E-05	3.70E-02	0.0148	2.75E-07	1.44E-03	0.012
cg25122233	0.0200	1.61E-05	3.70E-02	0.0225	4.43E-06	1.29E-02	0.018
cg18828927	0.0080	1.62E-05	3.70E-02	0.0072	3.41E-04	1.88E-01	0.006
cg08698721	0.0130	1.66E-05	3.74E-02	0.0130	1.37E-05	2.94E-02	0.012
cg22879098	-0.0300	1.67E-05	3.74E-02	-0.0290	7.75E-05	8.66E-02	-0.025
cg07448928	0.0070	1.67E-05	3.74E-02	0.0081	6.81E-06	1.76E-02	0.006
cg12443001	0.0100	1.67E-05	3.74E-02	0.0115	7.33E-07	3.16E-03	0.008
cg23458168	0.0290	1.76E-05	3.90E-02	0.0319	7.64E-07	3.25E-03	0.033
cg13547053	0.0100	1.83E-05	3.99E-02	0.0082	2.54E-04	1.65E-01	0.011
cg17345450	-0.0110	1.83E-05	3.99E-02	-0.0098	1.72E-04	1.32E-01	-0.014
cg23928512	-0.0110	1.83E-05	3.99E-02	-0.0076	2.99E-03	4.87E-01	-0.013
cg07553761	0.0040	1.94E-05	4.19E-02	0.0043	2.49E-05	4.41E-02	0.005
cg13459104	0.0030	1.95E-05	4.19E-02	0.0030	9.34E-05	9.52E-02	0.003
cg15893360	-0.0130	1.96E-05	4.19E-02	-0.0139	6.18E-06	1.65E-02	-0.012
cg02404974	0.0150	1.99E-05	4.23E-02	0.0159	1.09E-05	2.46E-02	0.015
cg04807108	0.0220	2.01E-05	4.24E-02	0.0228	1.91E-05	3.71E-02	0.026
cg22171758	0.0120	2.02E-05	4.24E-02	0.0131	8.25E-06	2.05E-02	0.01
cg03486379	0.0090	2.03E-05	4.24E-02	0.0088	1.60E-04	1.27E-01	0.008

Probe	Model1[b] Coeff	Model1[b] *P*-value	Model1[b] *q*-value	Model2[c] Coeff	Model2[c] *P*-value	Model2[c] *q*-value	Δ β-value[d]
cg07464358	0.0080	2.08E-05	4.30E-02	0.0073	5.79E-05	7.34E-02	0.011
cg19675142	0.0130	2.08E-05	4.30E-02	0.0127	8.15E-05	8.80E-02	0.013
cg08755703	0.0170	2.12E-05	4.36E-02	0.0181	9.89E-06	2.28E-02	0.022
cg20174893	-0.0100	2.16E-05	4.41E-02	-0.0110	2.05E-05	3.88E-02	-0.009
cg10788371	-0.0100	2.20E-05	4.47E-02	-0.0090	9.55E-05	9.64E-02	-0.009
cg06509837	-0.0260	2.22E-05	4.47E-02	-0.0205	1.06E-03	3.27E-01	-0.025
cg18209323	0.0160	2.23E-05	4.47E-02	0.0151	6.37E-05	7.66E-02	0.016
cg11064524	-0.0110	2.33E-05	4.65E-02	-0.0106	1.04E-04	1.03E-01	-0.013
cg03687532	0.0120	2.41E-05	4.77E-02	0.0117	4.94E-05	6.59E-02	0.015
cg26580869	-0.0190	2.41E-05	4.77E-02	-0.0172	4.57E-05	6.38E-02	-0.02
cg13455434	0.0260	2.44E-05	4.77E-02	0.0202	1.34E-03	3.59E-01	0.027
cg16944958	-0.0120	2.44E-05	4.77E-02	-0.0122	3.85E-05	5.76E-02	-0.01
cg26827653	0.0120	2.50E-05	4.85E-02	0.0147	1.42E-06	5.27E-03	0.012
cg19563365	0.0070	2.56E-05	4.94E-02	0.0070	2.89E-05	4.78E-02	0.007

Abbreviations: Chr: chromosome, coeff: coefficient

[a]FDR q<0.05 (Model1). [b]Model1: Methylation β-value = maternal smoking + infant's sex + infant's cleft status + batch effect + bisulfite conversion efficiency. [c]Model2: Methylation β-value = maternal smoking + infant's sex + infant's cleft status + batch effect + bisulfite conversion efficiency + maternal alcohol use + maternal education + maternal age at delivery + maternal folic acid supplement use + infant's birth weight + adjustment for 5 different cell type proportions using GSE35069. [d]β-value represents the ratio of methylated signal to total signal (methylated plus unmethylated). This shows the difference between the mean β-value of smokers and non-smokers.

[*]Meets bonferroni correction ($p<1.4x10^{-7}$).

Table S3. CpG probe annotation.

Probe	Nearest gene	Location[a]	Left gene[b]	Right gene[b]	Left gene: Direction[c]	Right gene: Direction[c]	Gene: Direction[c]	(Left) gene: Distance[d]	(Right) gene: Distance[d]
cg06415891	*HES5*	Intergenic	*HES5*	*LOC115110*	-	-	NA	5412	14263
cg20816361	*HES5*	Intergenic	*HES5*	*LOC115110*	-	-	NA	5416	14259
cg12443001	*HES5*	Intergenic	*HES5*	*LOC115110*	-	-	NA	5597	14078
cg08142858	*TTC34*	Intergenic	*TTC34*	*ACTRT2*	-	+	NA	12512	219304
cg15836046	*TTC34*	Intergenic	*TTC34*	*ACTRT2*	-	+	NA	61012	170804
cg15522425	*TTC34*	Intergenic	*TTC34*	*ACTRT2*	-	+	NA	86407	145409
cg15325070	*TTC34*	Intergenic	*TTC34*	*ACTRT2*	-	+	NA	86474	145342
cg22877366	*MIIP*	Intergenic	*MIIP*	*TNFRSF8*	+	+	NA	15562	15766
cg15963463	*RUNX3*	Intron	NA	NA	NA	NA	-	27235	38264
cg05656688	*RUNX3*	CDS	NA	NA	NA	NA	-	28086	37413
cg25189904	*GNG12*	Intergenic	*GNG12*	*LOC100133029*	-	+	NA	338	209519
cg26764244	*GNG12*	Intergenic	*GNG12*	*LOC100133029*	-	+	NA	356	209501
cg10399789	*GFI1*	Intron	NA	NA	NA	NA	-	5350	6765
cg09662411	*GFI1*	Intron	NA	NA	NA	NA	-	5814	6301
cg06338710	*GFI1*	CDS	NA	NA	NA	NA	-	5869	6246
cg18146737	*GFI1*	Intron	NA	NA	NA	NA	-	6382	5733
cg12876356	*GFI1*	Intron	NA	NA	NA	NA	-	6507	5608
cg18316974	*GFI1*	Intron	NA	NA	NA	NA	-	6717	5398
cg09935388	*GFI1*	Intron	NA	NA	NA	NA	-	7270	4845
cg14179389	*GFI1*	Intron	NA	NA	NA	NA	-	7643	4472
cg19675142	*LOC100132332*	Intron	NA	NA	NA	NA	+	12	1038
cg17442776	*TRNAQ8*	Intergenic	*LOC100505824*	*TRNAQ8*	+	-	NA	82852	18842
cg16517298	*GALNT2*	Intron	NA	NA	NA	NA	+	210218	4701
cg19727396	*GALNT2*	CDS	NA	NA	NA	NA	+	212229	2690

Probe	Nearest gene	Location[a]	Left gene[b]	Right gene[b]	Left gene: Direction[c]	Right gene: Direction[c]	Gene: Direction[c]	(Left) gene: Distance[d]	(Right) gene: Distance[d]
cg24591105	*GALNT2*	UTR	NA	NA	NA	NA	+	212269	2650
cg00589617	*GALNT2*	UTR	NA	NA	NA	NA	+	212387	2532
cg05697274	*GALNT2*	UTR	NA	NA	NA	NA	+	212421	2498
cg24250902	*GALNT2*	UTR	NA	NA	NA	NA	+	212591	2328
cg03144619	*GALNT2*	UTR	NA	NA	NA	NA	+	212712	2207
cg09368188	*KIF26B*	Intron	NA	NA	NA	NA	+	11731	536410
cg26119806	*KCNS3*	Intron	NA	NA	NA	NA	+	1627	52653
cg08026735	*VWA3B*	Intron	NA	NA	NA	NA	+	293	225522
cg18703066	*LOC284998*	UTR	NA	NA	NA	NA	-	441	10641
cg13547053	*OSBPL6*	Intron	NA	NA	NA	NA	+	125656	79296
cg22057874	*OSBPL6*	Intron	NA	NA	NA	NA	+	125658	79294
cg11641006	*RPS20P12*	Intergenic	*SPP2*	*RPS20P12*	+	+	NA	228098	24172
cg18096987	*VGLL4*	Intron	NA	NA	NA	NA	-	26329	138347
cg18722086	*KIAA2018*	CDS	NA	NA	NA	NA	-	12840	35421
cg07553761	*TRIM59*	Intergenic	*TRIM59*	*B3GAT3P1*	-	+	NA	351	2179
cg04098479	*WHSC2*	Intron	NA	NA	NA	NA	-	10406	16110
cg16449012	*FAM184B*	Intron	NA	NA	NA	NA	-	148184	1255
cg14349977	*LOC285441*	Intron	NA	NA	NA	NA	-	12178	202782
cg18753646	*LOC285441*	Intron	NA	NA	NA	NA	-	12286	202674
cg17924476	*AHRR*	Intron	NA	NA	NA	NA	+	19503	114612
cg23067299	*AHRR*	Intron	NA	NA	NA	NA	+	19616	114499
cg08606254	*AHRR*	Intron	NA	NA	NA	NA	+	19678	114437
cg12806681	*AHRR*	Intron	NA	NA	NA	NA	+	64103	70012
cg03991871	*AHRR*	Intron	NA	NA	NA	NA	+	64156	69959
cg23916896	*AHRR*	Intron	NA	NA	NA	NA	+	64513	69602
cg11902777	*AHRR*	Intron	NA	NA	NA	NA	+	64552	69563

Probe	Nearest gene	Location[a]	Left gene[b]	Right gene[b]	Left gene: Direction[c]	Right gene: Direction[c]	Gene: Direction[c]	(Left) gene: Distance[d]	(Right) gene: Distance[d]
cg05575921	*AHRR*	Intron	NA	NA	NA	NA	+	69087	65028
cg14817490	*AHRR*	Intron	NA	NA	NA	NA	+	88629	45486
cg21161138	*AHRR*	Intron	NA	NA	NA	NA	+	95069	39046
cg22937882	*AHRR*	Intron	NA	NA	NA	NA	+	101483	32632
cg07448928	*AHRR*	CDS	NA	NA	NA	NA	+	123464	10651
cg10841124	*AHRR*	Intron	NA	NA	NA	NA	+	128983	5132
cg26954197	*AHRR*	UTR	NA	NA	NA	NA	+	132525	1590
cg22879098	*TPPP*	Intron	NA	NA	NA	NA	-	12868	20665
cg01952185	*TIFAB*	Intergenic	*TIFAB*	*NEUROG1*	-	-	NA	25124	56759
cg11429111	*TIFAB*	Intergenic	*TIFAB*	*NEUROG1*	-	-	NA	25240	56643
cg25383593	*TIFAB*	Intergenic	*TIFAB*	*NEUROG1*	-	-	NA	39731	42152
cg25415695	*TIFAB*	Intergenic	*TIFAB*	*NEUROG1*	-	-	NA	39947	41936
cg04800195	*ZFP62*	Intron	NA	NA	NA	NA	-	13293	382
cg26889659	*EXOC2*	Intron	NA	NA	NA	NA	-	198952	9019
cg18694169	*NKAPL*	Intergenic	*ZKSCAN4*	*NKAPL*	-	+	NA	7077	19
cg10253847	*NKAPL*	Intergenic	*ZKSCAN4*	*NKAPL*	-	+	NA	7083	13
cg01031101	*NKAPL*	Intergenic	*ZKSCAN4*	*NKAPL*	-	+	NA	7089	7
cg09523275	*NKAPL*	Intergenic	*ZKSCAN4*	*NKAPL*	-	+	NA	7091	5
cg18675097	*NKAPL*	UTR	NA	NA	NA	NA	+	29	1609
cg18828927	*OR14J1*	CDS	NA	NA	NA	NA	+	796	169
cg14027333	*PRRT1*	UTR	NA	NA	NA	NA	-	177	3403
cg02404974	*LOC100505711*	Intergenic	*LOC100505711*	*LOC100505730*	-	-	NA	25472	115338
cg14086013	*MDFI*	Intergenic	*FOXP4*	*MDFI*	+	+	NA	34618	1455
cg03354508	*YAP1P1*	Intergenic	*YAP1P1*	*SAMD5*	+	+	NA	40081	60330
cg17345450	*SDIM1*	Intergenic	*PDE10A*	*SDIM1*	-	-	NA	184354	47392
cg00794911	*SDIM1*	Intergenic	*PDE10A*	*SDIM1*	-	-	NA	184948	46798

Probe	Nearest gene	Location[a]	Left gene[b]	Right gene[b]	Left gene: Direction[c]	Right gene: Direction[c]	Gene: Direction[c]	(Left) gene: Distance[d]	(Right) gene: Distance[d]
cg18132363	*SDIM1*	Intergenic	*PDE10A*	*SDIM1*	-	-	NA	184988	46758
cg11625488	*LOC100505903*	Intergenic	*C6orf208*	*LOC100505903*	+	+	NA	200588	72339
cg10312186	*LOC100505903*	Intergenic	*C6orf208*	*LOC100505903*	+	+	NA	200614	72313
cg16944958	*LOC100505903*	Intergenic	*C6orf208*	*LOC100505903*	+	+	NA	200993	71934
cg07249149	*C7orf50*	Intergenic	*CYP2W1*	*C7orf50*	+	-	NA	6087	1260
cg07104557	*C7orf50*	UTR	NA	NA	NA	NA	-	30	141240
cg19089201	*MYO1G*	UTR	NA	NA	NA	NA	-	27	16417
cg22132788	*MYO1G*	CDS	NA	NA	NA	NA	-	226	16218
cg04180046	*MYO1G*	Intron	NA	NA	NA	NA	-	476	15968
cg12803068	*MYO1G*	Intron	NA	NA	NA	NA	-	659	15785
cg16459265	*C7orf40*	Intron	NA	NA	NA	NA	-	2453	1179
cg04598670	*LOC100507468*	Intergenic	*LOC100419458*	*LOC100507468*	+	-	NA	591057	363472
cg18209323	*SAMD9*	Intergenic	*RN7SL7P*	*SAMD9*	-	-	NA	72238	55948
cg20174893	*MGC72080*	Intron	NA	NA	NA	NA	-	5018	712
cg07464358	*RABL5*	Intergenic	*LOC100506507*	*RABL5*	+	-	NA	7739	4284
cg25949550	*CNTNAP2*	Intron	NA	NA	NA	NA	+	853	2303784
cg15578140	*CNTNAP2*	Intron	NA	NA	NA	NA	+	1904656	399981
cg00433296	*SLC39A14*	Intergenic	*PIWIL2*	*SLC39A14*	+	+	NA	10268	910
cg13455434	*RBPMS*	Intron	NA	NA	NA	NA	+	1986	185804
cg02892925	*LOC100505501*	UTR	NA	NA	NA	NA	+	1149	1471
cg07211044	*LOC100505501*	UTR	NA	NA	NA	NA	+	1206	1414
cg18794473	*ZFHX4*	CDS	NA	NA	NA	NA	+	24179	161827
cg04640109	*ZFPM2*	Intergenic	*RPL17P32*	*ZFPM2*	-	+	NA	29580	974
cg11064524	*EXT1*	Intron	NA	NA	NA	NA	-	63527	248929
cg13459104	*LOC158435*	Intron	NA	NA	NA	NA	+	21600	14473
cg01825213	*HSD17B3*	Intergenic	*EIF4BP3*	*HSD17B3*	+	-	NA	69830	17624

Probe	Nearest gene	Location[a]	Left gene[b]	Right gene[b]	Left gene: Direction[c]	Right gene: Direction[c]	Gene: Direction[c]	(Left) gene: Distance[d]	(Right) gene: Distance[d]
cg13784312	*RAPGEF1*	Intron	NA	NA	NA	NA	-	156908	3860
cg13822849	*OLFM1*	Intron	NA	NA	NA	NA	+	32668	13273
cg20344448	*FRMD4A*	Intron	NA	NA	NA	NA	-	686725	435
cg11813497	*FRMD4A*	Intergenic	*FRMD4A*	*MIR4293*	-	-	NA	13	52320
cg25464840	*FRMD4A*	Intergenic	*FRMD4A*	*MIR4293*	-	-	NA	44	52289
cg15507334	*FRMD4A*	Intergenic	*FRMD4A*	*MIR4293*	-	-	NA	47	52286
cg03486379	*PARD3*	Intergenic	*RPL23P11*	*PARD3*	+	-	NA	237605	7232
cg24239148	*C10orf107*	UTR	NA	NA	NA	NA	+	122	103250
cg06813382	*LOC439990*	Intergenic	*ANXA11*	*LOC439990*	-	+	NA	1867	271
cg08626436	*BMPR1A*	Intron	NA	NA	NA	NA	+	38943	129606
cg09488203	*O3FAR1*	Intron	NA	NA	NA	NA	+	1462	21945
cg05593775	*PDZD7*	CDS	NA	NA	NA	NA	-	11303	12171
cg18621256	*EMX2OS*	Intergenic	*PDZD8*	*EMX2OS*	-	-	NA	86507	22360
cg10673740	*BAG3*	Intron	NA	NA	NA	NA	+	944	25505
cg22043296	*LSP1*	UTR	NA	NA	NA	NA	+	17939	21354
cg19631472	*LDHA*	Intergenic	*MIR3159*	*LDHA*	+	+	NA	6234	295
cg10788371	*LRRC32*	UTR	NA	NA	NA	NA	-	12472	751
cg09063262	*TMEM126A*	Intergenic	*TMEM126B*	*TMEM126A*	+	+	NA	10923	457
cg19563365	*PRSS23*	Intergenic	*ME3*	*PRSS23*	-	+	NA	124721	3092
cg02356218	*OR8B9P*	Intergenic	*OR8B9P*	*OR8B10P*	-	-	NA	589	34158
cg26827653	*MGC39545*	UTR	NA	NA	NA	NA	+	1315	167
cg10520740	*CACNA2D4*	Intron	NA	NA	NA	NA	-	51675	75072
cg22171758	*CALCOCO1*	Intergenic	*CALCOCO1*	*HOXC13*	-	+	NA	24826	186443
cg25122233	*HOXC4*	Intron	NA	NA	NA	NA	+	1864	37308
cg03453449	*USP44*	UTR	NA	NA	NA	NA	-	34241	143
cg25632883	*SSH1*	Intron	NA	NA	NA	NA	-	73972	921

Probe	Nearest gene	Location[a]	Left gene[b]	Right gene[b]	Left gene: Direction[c]	Right gene: Direction[c]	Gene: Direction[c]	(Left) gene: Distance[d]	(Right) gene: Distance[d]
cg24698536	*SSH1*	Intron	NA	NA	NA	NA	-	74049	844
cg12492906	*SSH1*	Intron	NA	NA	NA	NA	-	74079	814
cg00029284	*CUX2*	Intron	NA	NA	NA	NA	+	259375	57155
cg26103179	*SRRM4*	Intron	NA	NA	NA	NA	+	172364	9192
cg12160087	*CCDC64*	UTR	NA	NA	NA	NA	+	104338	313
cg15893360	*PXN*	Intron	NA	NA	NA	NA	-	52111	3202
cg08698721	*MEG3*	Intron	NA	NA	NA	NA	+	1702	33216
cg04291079	*MEG3*	Intron	NA	NA	NA	NA	+	1985	32933
cg26554302	*IGHV1OR15-3*	Intergenic	*IGHV1OR15-3*	*LOC642131*	-	-	NA	833	4128
cg03796381	*COX6CP4*	Intergenic	*LOC100507480*	*COX6CP4*	+	+	NA	308303	214098
cg10078511	*THBS1*	Intergenic	*C15orf54*	*THBS1*	+	+	NA	324984	1248
cg23849826	*THBS1*	Intergenic	*C15orf54*	*THBS1*	+	+	NA	325138	1094
cg16231923	*NPTN*	Intron	NA	NA	NA	NA	-	71290	2119
cg00213123	*CYP1A1*	Intergenic	*CYP1A1*	*CYP1A2*	-	+	NA	1193	22114
cg05549655	*CYP1A1*	Intergenic	*CYP1A1*	*CYP1A2*	-	+	NA	1266	22041
cg17852385	*CYP1A1*	Intergenic	*CYP1A1*	*CYP1A2*	-	+	NA	1311	21996
cg13570656	*CYP1A1*	Intergenic	*CYP1A1*	*CYP1A2*	-	+	NA	1319	21988
cg12101586	*CYP1A1*	Intergenic	*CYP1A1*	*CYP1A2*	-	+	NA	1326	21981
cg22549041	*CYP1A1*	Intergenic	*CYP1A1*	*CYP1A2*	-	+	NA	1374	21933
cg11924019	*CYP1A1*	Intergenic	*CYP1A1*	*CYP1A2*	-	+	NA	1406	21901
cg18092474	*CYP1A1*	Intergenic	*CYP1A1*	*CYP1A2*	-	+	NA	1425	21882
cg26516004	*CYP1A1*	Intergenic	*CYP1A1*	*CYP1A2*	-	+	NA	1499	21808
cg01359532	*CYP1A2*	Intron	NA	NA	NA	NA	+	3942	3815
cg04807108	*RWDD1P1*	Intergenic	*RWDD1P1*	*IREB2*	+	+	NA	22391	32585
cg12937151	*AGBL1*	Intergenic	*AGBL1*	*LINC00052*	+	+	NA	185943	361934
cg04459939	*MESP1*	Intergenic	*WDR93*	*MESP1*	+	-	NA	4272	1957

Probe	Nearest gene	Location[a]	Left gene[b]	Right gene[b]	Left gene: Direction[c]	Right gene: Direction[c]	Gene: Direction[c]	(Left) gene: Distance[d]	(Right) gene: Distance[d]
cg23335299	*MESP1*	Intergenic	*WDR93*	*MESP1*	+	-	NA	4887	1342
cg13834112	*ANPEP*	Intergenic	*ANPEP*	*C15orf38-AP3S2*	-	-	NA	3567	12192
cg26804595	*FBXL16*	Intron	NA	NA	NA	NA	-	7081	6244
cg13928411	*VWA3A*	Intergenic	*C16orf52*	*VWA3A*	+	+	NA	7816	72
cg09743950	*ITGAM*	CDS	NA	NA	NA	NA	+	70587	2338
cg00253658	*FTO*	Intergenic	*FTO*	*IRX3*	+	-	NA	62117	106716
cg26681628	*FTO*	Intergenic	*FTO*	*IRX3*	+	-	NA	62171	106662
cg03687532	*FTO*	Intergenic	*FTO*	*IRX3*	+	-	NA	79979	88854
cg03177666	*IRX3*	Intergenic	*IRX3*	*CRNDE*	-	-	NA	44725	587672
cg08755703	*IRX3*	Intergenic	*IRX3*	*CRNDE*	-	-	NA	74611	557786
cg24173182	*HIC1*	CDS	NA	NA	NA	NA	+	2893	1695
cg05568941	*GJD3*	UTR	NA	NA	NA	NA	-	1009	3032
cg06949812	*GJD3*	UTR	NA	NA	NA	NA	-	1089	2952
cg11096905	*AXIN2*	Intron	NA	NA	NA	NA	-	10886	22173
cg23928512	*ASPSCR1*	Intron	NA	NA	NA	NA	+	34766	5090
cg23664708	*SLC16A3*	UTR	NA	NA	NA	NA	+	10621	472
cg06509837	*HCN2*	Intron	NA	NA	NA	NA	+	21062	6204
cg26580869	*HCN2*	Intron	NA	NA	NA	NA	+	21613	5653
cg02010738	*MOB3A*	Intron	NA	NA	NA	NA	-	24431	803
cg08097581	*AKAP8L*	Intron	NA	NA	NA	NA	-	37730	1244
cg26652413	*CPAMD8*	CDS	NA	NA	NA	NA	-	3295	130568
cg23458168	*ZNF536*	Intron	NA	NA	NA	NA	+	1539	184098
cg16547579	*SLC23A2*	Intron	NA	NA	NA	NA	-	121331	27812
cg20244340	*SLC24A3*	Intron	NA	NA	NA	NA	+	699	509552
cg11868728	*FOXA2*	Intergenic	*FOXA2*	*KRT18P3*	-	-	NA	2015	145255
cg20274009	*TPM3P2*	Intergenic	*CHMP4B*	*TPM3P2*	+	-	NA	8673	7535

Probe	Nearest gene	Location[a]	Left gene[b]	Right gene[b]	Left gene: Direction[c]	Right gene: Direction[c]	Gene: Direction[c]	(Left) gene: Distance[d]	(Right) gene: Distance[d]
cg07339236	*ATP9A*	Intron	NA	NA	NA	NA	-	99176	72418
cg20643029	*CLDN14*	Intron	NA	NA	NA	NA	-	82125	33823
cg12426652	*CLDN14*	Intron	NA	NA	NA	NA	-	82137	33811
cg04478905	*CLDN14*	Intron	NA	NA	NA	NA	-	82172	33776
cg26441486	*CRELD2*	Intron	NA	NA	NA	NA	+	5017	3886

Abbreviations: CDS: coding DNA sequence, NA: not applicable, UTR: untranslated region

[a]If the CpG is listed as being part of the CDS or UTR, this annotation could change depending on the transcript used as the reference. [b]For only intergenic CpGs, the neighboring gene to the left or right is listed. [c]The '+' and '-' indicate if the gene is transcribed on the forward or reverse strand, respectively. [d]Distance (basepairs) to the UTR. If intergenic, the minimum distance to the left or right neighboring gene is listed. If within the gene body, distance to the start of the 5'UTR and end of 3'UTR is listed.

Table S4. CpG probe annotation.

Probe	Chr	Basepair[a]	Distance to TSS[b]	Closest TSS Gene[c]	CpG Island[d]
cg06415891	1	2467096	-5412	*HES5*	Island
cg20816361	1	2467100	-5416	*HES5*	Island
cg12443001	1	2467281	-5597	*HES5*	Shore
cg08142858	1	2718742	-12512	*TTC34*	Shore
cg15836046	1	2767242	-61012	*TTC34*	Non-island
cg15522425	1	2792637	-86407	*TTC34*	Non-island
cg15325070	1	2792704	-86474	*TTC34*	Non-island
cg22877366	1	12107668	-15765	*TNFRSF8*	Non-island
cg15963463	1	25253237	3533	*RUNX3*	Shelf
cg05656688	1	25254088	2682	*RUNX3*	Shore
cg25189904	1	68299493	-338	*GNG12*	Shore
cg26764244	1	68299511	-356	*GNG12*	Shore
cg10399789	1	92945668	3688	*GFI1*	Shore
cg09662411	1	92946132	3224	*GFI1*	Island
cg06338710	1	92946187	3169	*GFI1*	Island
cg18146737	1	92946700	2656	*GFI1*	Island
cg12876356	1	92946825	2531	*GFI1*	Island
cg18316974	1	92947035	2321	*GFI1*	Island
cg09935388	1	92947588	1768	*GFI1*	Island
cg14179389	1	92947961	1395	*GFI1*	Island
cg19675142	1	116107015	-77558	*VANGL1*	Non-island
cg17442776	1	147718540	-488	*TRNA_Asn*	Island
cg16517298	1	230413174	15559	*GALNT2*	Shelf
cg19727396	1	230415185	17570	*GALNT2*	Shore
cg24591105	1	230415225	17610	*GALNT2*	Shore

Probe	Chr	Basepair[a]	Distance to TSS[b]	Closest TSS Gene[c]	CpG Island[d]
cg00589617	1	230415343	17728	*GALNT2*	Island
cg05697274	1	230415377	17762	*GALNT2*	Island
cg24250902	1	230415547	17932	*GALNT2*	Island
cg03144619	1	230415668	18053	*GALNT2*	Island
cg09368188	1	245330018	11732	*KIF26B*	Non-island
cg26119806	2	18061572	1628	*KCNS3*	Shore
cg08026735	2	98703888	294	*VWA3B*	Island
cg18703066	2	105363536	8677	*LOC284998*	Non-island
cg13547053	2	179184864	-106	*OSBPL6*	Non-island
cg22057874	2	179184866	-104	*OSBPL6*	Non-island
cg11641006	2	235213874	149479	*AX748264*	Non-island
cg18096987	3	11623873	-37	*VGLL4*	Non-island
cg18722086	3	113380072	9008	*KIAA2018*	Non-island
cg07553761	3	160167977	-351	*IFT80*	Island
cg04098479	4	1994849	-878	*WHSC2*	Island
cg16449012	4	17781880	1255	*FAM184B*	Shore
cg14349977	4	187219430	32313	*F11*	Island
cg18753646	4	187219538	32421	*F11*	Island
cg17924476	5	323794	1093	*AHRR*	Shore
cg23067299	5	323907	1206	*AHRR*	Shore
cg08606254	5	323969	1268	*AHRR*	Shore
cg12806681	5	368394	45693	*AHRR*	Shore
cg03991871	5	368447	45746	*AHRR*	Shore
cg23916896	5	368804	46103	*AHRR*	Shore
cg11902777	5	368843	46142	*AHRR*	Shore
cg05575921	5	373378	-47656	*AHRR*	Shore
cg14817490	5	392920	-28114	*AHRR*	Non-island

Probe	Chr	Basepair[a]	Distance to TSS[b]	Closest TSS Gene[c]	CpG Island[d]
cg21161138	5	399360	-21674	*AHRR*	Non-island
cg22937882	5	405774	-15260	*AHRR*	Non-island
cg07448928	5	427755	-4384	*AHRR*	Non-island
cg10841124	5	433274	1135	*AHRR*	Non-island
cg26954197	5	436816	4677	*AHRR*	Non-island
cg22879098	5	672845	6048	*TPPP*	Non-island
cg01952185	5	134813213	-25124	*TIFAB*	Non-island
cg11429111	5	134813329	-25240	*TIFAB*	Non-island
cg25383593	5	134827820	-39731	*TIFAB*	Shore
cg25415695	5	134828036	-39947	*TIFAB*	Shore
cg04800195	5	180287904	382	*ZFP62*	Island
cg26889659	6	684090	9019	*EXOC2*	Non-island
cg18694169	6	28227079	-18	*NKAPL*	Island
cg10253847	6	28227085	-12	*NKAPL*	Island
cg01031101	6	28227091	-6	*NKAPL*	Island
cg09523275	6	28227093	-4	*NKAPL*	Island
cg18675097	6	28227127	30	*NKAPL*	Island
cg18828927	6	29275263	797	*OR14J1*	Non-island
cg14027333	6	32116317	3383	*PRRT1*	Shore
cg02404974	6	41376295	72768	*NCR2*	Shore
cg14086013	6	41604740	-1454	*MDFI*	Island
cg03354508	6	147769498	-60329	*SAMD5*	Non-island
cg17345450	6	166259938	140046	*AK090688*	Non-island
cg00794911	6	166260532	139452	*AK090688*	Non-island
cg18132363	6	166260572	139412	*AK090688*	Non-island
cg11625488	6	170403557	168100	*LOC154449*	Shore
cg10312186	6	170403583	168074	*LOC154449*	Shore

Probe	Chr	Basepair[a]	Distance to TSS[b]	Closest TSS Gene[c]	CpG Island[d]
cg16944958	6	170403962	167695	*LOC154449*	Shore
cg07249149	7	1035363	12529	*CYP2W1*	Non-island
cg07104557	7	1036653	13819	*CYP2W1*	Shelf
cg19089201	7	45002287	6699	*BC041623*	Island
cg22132788	7	45002486	6500	*BC041623*	Island
cg04180046	7	45002736	6250	*BC041623*	Island
cg12803068	7	45002919	6067	*BC041623*	Shore
cg16459265	7	45025080	29	*SNORA9*	Shore
cg04598670	7	68697651	168638	*U6*	Non-island
cg18209323	7	92672878	62532	*SAMD9*	Island
cg20174893	7	97600926	712	*MGC72080*	Shore
cg07464358	7	100952364	744	*BC032716*	Non-island
cg25949550	7	145814306	854	*CNTNAP2*	Shore
cg15578140	7	147718109	-79507	*MIR548F3*	Non-island
cg00433296	8	22223852	-909	*SLC39A14*	Shore
cg13455434	8	30243930	-1013	*AK057935*	Shore
cg02892925	8	60032926	-1159	*TOX*	Shore
cg07211044	8	60032983	-1216	*TOX*	Shore
cg18794473	8	77617694	1417	*ZFHX4*	Non-island
cg04640109	8	106330173	-973	*ZFPM2*	Shore
cg11064524	8	118875129	225369	*AK025288*	Non-island
cg13459104	9	98849721	-7558	*LOC158435*	Non-island
cg01825213	9	98979965	84469	*HSD17B3*	Shore
cg13784312	9	134609065	3860	*RAPGEF1*	Non-island
cg13822849	9	137999757	2272	*OLFM1*	Shelf
cg20344448	10	14372431	435	*FRMD4A*	Non-island
cg11813497	10	14372879	-13	*FRMD4A*	Non-island

Probe	Chr	Basepair[a]	Distance to TSS[b]	Closest TSS Gene[c]	CpG Island[d]
cg25464840	10	14372910	-44	*FRMD4A*	Non-island
cg15507334	10	14372913	-47	*FRMD4A*	Non-island
cg03486379	10	34391256	-7552	*AX765719*	Non-island
cg24239148	10	63422841	123	*C10orf107*	Island
cg06813382	10	81967195	-270	*LOC439990*	Island
cg08626436	10	88555339	38944	*BMPR1A*	Non-island
cg09488203	10	95327884	1463	*O3FAR1*	Shore
cg05593775	10	102778743	12171	*PDZD7*	Island
cg18621256	10	119221444	80480	*EMX2OS*	Non-island
cg10673740	10	121411826	945	*BAG3*	Island
cg22043296	11	1892139	-14	*LSP1*	Island
cg19631472	11	18415641	-294	*LDHA*	Shore
cg10788371	11	76381040	4	*LRRC32*	Shore
cg09063262	11	85358506	-456	*TMEM126A*	Shore
cg19563365	11	86508399	-3091	*PRSS23*	Shelf
cg02356218	11	124352048	-41067	*OR8B8*	Non-island
cg26827653	11	125366540	-334	*FEZ1*	Shore
cg10520740	12	1952798	10412	*CACNA2D4*	Non-island
cg22171758	12	54146133	-24826	*CALCOCO1*	Shore
cg25122233	12	54412506	1865	*HOXC5*	Shore
cg03453449	12	95945120	143	*USP44*	Shelf
cg25632883	12	109250438	921	*SSH1*	Shore
cg24698536	12	109250515	844	*SSH1*	Shore
cg12492906	12	109250545	814	*SSH1*	Shore
cg00029284	12	111731203	70028	*FAM109A*	Non-island
cg26103179	12	119591664	-24930	*HSPB8*	Shore
cg12160087	12	120531986	22561	*CCDC64*	Shelf

Probe	Chr	Basepair[a]	Distance to TSS[b]	Closest TSS Gene[c]	CpG Island[d]
cg15893360	12	120700361	3213	*PXN*	Shelf
cg08698721	14	101294147	-1223	*MEG3*	Island
cg04291079	14	101294430	-940	*MEG3*	Island
cg26554302	15	22467326	-18540	*AJ004954*	Non-island
cg03796381	15	36650033	-221778	*C15orf41*	Non-island
cg10078511	15	39872032	-1247	*THBS1*	Shore
cg23849826	15	39872186	-1093	*THBS1*	Shore
cg16231923	15	73923634	2119	*NPTN*	Shore
cg00213123	15	75019070	-1193	*CYP1A1*	Island
cg05549655	15	75019143	-1266	*CYP1A1*	Island
cg17852385	15	75019188	-1311	*CYP1A1*	Island
cg13570656	15	75019196	-1319	*CYP1A1*	Island
cg12101586	15	75019203	-1326	*CYP1A1*	Island
cg22549041	15	75019251	-1374	*CYP1A1*	Island
cg11924019	15	75019283	-1406	*CYP1A1*	Island
cg18092474	15	75019302	-1425	*CYP1A1*	Island
cg26516004	15	75019376	-1499	*CYP1A1*	Shore
cg01359532	15	75045126	3943	*CYP1A2*	Non-island
cg04807108	15	78697933	-32584	*IREB2*	Non-island
cg12937151	15	87758226	-361933	*LINC00052*	Non-island
cg04459939	15	90291141	3399	*MESP1*	Shelf
cg23335299	15	90291756	2784	*MESP1*	Shore
cg13834112	15	90361639	-3567	*ANPEP*	Shelf
cg26804595	16	749581	-3556	*FBXL16*	Shore
cg13928411	16	22103791	-71	*VWA3A*	Non-island
cg09743950	16	31341875	5314	*DL489986*	Shore
cg00253658	16	54210496	109882	*IRX3*	Non-island

Probe	Chr	Basepair[a]	Distance to TSS[b]	Closest TSS Gene[c]	CpG Island[d]
cg26681628	16	54210550	109828	*IRX3*	Non-island
cg03687532	16	54228358	92020	*IRX3*	Island
cg03177666	16	54365103	-44725	*IRX3*	Non-island
cg08755703	16	54394989	-74611	*IRX3*	Non-island
cg24173182	17	1961286	1302	*HIC1*	Island
cg05568941	17	38517913	1007	*GJD3*	Shore
cg06949812	17	38517993	1087	*GJD3*	Shore
cg11096905	17	63535567	19287	*AXIN2*	Shore
cg23928512	17	79970192	10581	*STRA13*	Shelf
cg23664708	17	80196903	3254	*SLC16A3*	Shore
cg06509837	19	610955	21063	*HCN2*	Shore
cg26580869	19	611506	21614	*HCN2*	Island
cg02010738	19	2095466	803	*MOB3A*	Island
cg08097581	19	15528589	1244	*AKAP8L*	Shore
cg26652413	19	17007057	727	*CPAMD8*	Shore
cg23458168	19	30864867	1540	*ZNF536*	Shore
cg16547579	20	4954333	27812	*SLC23A2*	Non-island
cg20244340	20	19193989	700	*SLC24A3*	Shore
cg11868728	20	22568116	-2015	*FOXA2*	Shore
cg20274009	20	32450846	51737	*CHMP4B*	Shore
cg07339236	20	50312490	1552	*ATP9A*	Non-island
cg20643029	21	37915044	-146	*CLDN14*	Non-island
cg12426652	21	37915056	-158	*CLDN14*	Non-island
cg04478905	21	37915091	-193	*CLDN14*	Non-island
cg26441486	22	50317300	5018	*CRELD2*	Shore

Abbreviations: Chr: chromosome, TSS: transcription start site

[a]Physical location in basepairs (Human genome build GRCh37/hg19). [b]Distance in basepairs to the closest TSS (Price et al. 2013). [c]Gene with the closest TSS (Price et al. 2013). [d]CpG island annotation based on Illumina's 450K manifest file v1.2.

Table S5. Distribution of differentially methylated CpG sites by genomic region [n (%)].[a]

Description	Non-Island	Island	Shelf	Shore	P-value[b]
**Genome-wide significant CpG sites (FDR q<0.05)**					
Decreased methylation (N=80)	24 (30.0)	16 (20.0)	6 (7.5)	34 (42.5)	0.003*
Increased methylation (N=105)	36 (34.3)	33 (31.4)	5 (4.8)	31 (29.5)	0.061
All (N=185)	60 (32.4)	49 (26.5)	11 (5.9)	65 (35.1)	0.002*
**Illumina Human Methylation 450 BeadChip CpG sites**					
Decreased methylation (N=175106)	63936 (36.5)	50440 (28.8)	17522 (10.0)	43208 (24.7)	NA
Increased methylation (N=182214)	72959 (40.0)	47108 (25.9)	19620 (10.8)	42527 (23.3)	NA
All (N=357320)	136895 (38.3)	97548 (27.3)	37142 (10.4)	85735 (24.0)	NA

Abbreviations: NA: not applicable

[a]Annotation based on Illumina's 450K manifest file v1.2. [b]Chi-square test; Illumina Human Methylation 450 BeadChip used as the reference.

*P-value < 0.05

Table S6. Genome-wide significant CpG sites: dose-response evaluation by maternal smoking status.[a]

Probe	Non-active[b]	Light[b]	Moderate / Heavy[b]	Coeff[c]	*P*-value[c]	*q*-value[c]	Dose-response[d]
cg06415891	0.699 ± 0.002	0.714 ± 0.005	0.72 ± 0.005	0.0121	1.13E-06	4.77E-03	Yes
cg20816361	0.734 ± 0.002	0.746 ± 0.004	0.752 ± 0.004	0.0103	8.91E-07	4.02E-03	Yes
cg12443001	0.7 ± 0.001	0.712 ± 0.003	0.704 ± 0.003	0.0045	1.58E-03	4.62E-01	No
cg08142858	0.789 ± 0.001	0.794 ± 0.003	0.793 ± 0.002	0.0045	8.36E-05	1.16E-01	No
cg15836046	0.884 ± 0.001	0.89 ± 0.002	0.889 ± 0.001	0.0035	3.22E-06	1.15E-02	No
cg15522425	0.604 ± 0.002	0.616 ± 0.004	0.62 ± 0.003	0.0084	1.92E-07	1.18E-03	Yes
cg15325070	0.781 ± 0.002	0.793 ± 0.003	0.804 ± 0.003	0.0116	3.24E-11	3.73E-07	Yes
cg22877366	0.391 ± 0.002	0.369 ± 0.005	0.376 ± 0.005	-0.0105	2.09E-04	1.97E-01	No
cg15963463	0.334 ± 0.002	0.326 ± 0.004	0.327 ± 0.004	-0.0062	3.59E-05	7.49E-02	No
cg05656688	0.783 ± 0.002	0.766 ± 0.004	0.77 ± 0.004	-0.0073	1.41E-04	1.57E-01	No
cg25189904	0.598 ± 0.002	0.577 ± 0.005	0.582 ± 0.004	-0.0109	2.14E-07	1.25E-03	No
cg26764244	0.311 ± 0.002	0.291 ± 0.005	0.285 ± 0.005	-0.0143	3.98E-10	4.07E-06	Yes
cg10399789	0.868 ± 0.002	0.856 ± 0.004	0.838 ± 0.005	-0.0113	1.25E-12	1.59E-08	Yes
cg09662411	0.809 ± 0.003	0.783 ± 0.006	0.748 ± 0.008	-0.0241	9.84E-26	3.62E-21	Yes
cg06338710	0.895 ± 0.002	0.877 ± 0.005	0.851 ± 0.006	-0.0162	4.33E-29	2.28E-24	Yes
cg18146737	0.897 ± 0.004	0.85 ± 0.01	0.795 ± 0.012	-0.0464	2.80E-41	3.44E-36	Yes
cg12876356	0.831 ± 0.004	0.78 ± 0.009	0.729 ± 0.01	-0.0478	5.92E-40	4.36E-35	Yes
cg18316974	0.94 ± 0.003	0.912 ± 0.006	0.88 ± 0.008	-0.0231	2.19E-36	1.34E-31	Yes
cg09935388	0.827 ± 0.004	0.765 ± 0.009	0.72 ± 0.009	-0.0569	2.18E-41	3.44E-36	Yes
cg14179389	0.307 ± 0.004	0.255 ± 0.008	0.229 ± 0.008	-0.0418	1.99E-23	6.11E-19	Yes
cg19675142	0.51 ± 0.002	0.521 ± 0.004	0.525 ± 0.004	0.0085	6.01E-06	1.93E-02	Yes
cg17442776	0.215 ± 0.001	0.223 ± 0.002	0.223 ± 0.002	0.0052	1.91E-05	4.67E-02	Yes
cg16517298	0.479 ± 0.004	0.44 ± 0.007	0.464 ± 0.008	-0.0124	8.55E-04	3.67E-01	No
cg19727396	0.606 ± 0.002	0.589 ± 0.005	0.583 ± 0.005	-0.0112	1.27E-05	3.46E-02	Yes
cg24591105	0.47 ± 0.003	0.445 ± 0.005	0.443 ± 0.006	-0.0144	1.68E-07	1.06E-03	Yes

Probe	Non-active[b]	Light[b]	Moderate / Heavy[b]	Coeff[c]	*P*-value[c]	*q*-value[c]	Dose-response[d]
cg00589617	0.345 ± 0.003	0.318 ± 0.006	0.315 ± 0.007	-0.0171	3.78E-07	2.14E-03	Yes
cg05697274	0.408 ± 0.003	0.384 ± 0.006	0.384 ± 0.005	-0.0151	5.44E-08	3.85E-04	Yes
cg24250902	0.303 ± 0.003	0.276 ± 0.007	0.27 ± 0.007	-0.0192	2.54E-08	1.91E-04	Yes
cg03144619	0.431 ± 0.003	0.396 ± 0.007	0.391 ± 0.008	-0.0214	1.64E-08	1.28E-04	Yes
cg09368188	0.497 ± 0.003	0.519 ± 0.006	0.535 ± 0.007	0.0175	1.45E-08	1.16E-04	Yes
cg26119806	0.66 ± 0.002	0.645 ± 0.004	0.641 ± 0.004	-0.0108	4.11E-07	2.26E-03	Yes
cg08026735	0.577 ± 0.001	0.589 ± 0.003	0.588 ± 0.003	0.0065	2.95E-05	6.71E-02	No
cg18703066	0.959 ± 0.001	0.955 ± 0.002	0.955 ± 0.002	-0.0035	3.52E-11	3.93E-07	Yes
cg13547053	0.845 ± 0.001	0.856 ± 0.003	0.856 ± 0.003	0.006	4.46E-05	8.42E-02	No
cg22057874	0.596 ± 0.002	0.608 ± 0.003	0.609 ± 0.003	0.0073	3.26E-05	7.19E-02	No
cg11641006	0.707 ± 0.002	0.722 ± 0.004	0.72 ± 0.004	0.0092	1.71E-05	4.25E-02	No
cg18096987	0.501 ± 0.002	0.48 ± 0.004	0.495 ± 0.004	-0.0084	4.55E-05	8.54E-02	No
cg18722086	0.919 ± 0.001	0.923 ± 0.001	0.925 ± 0.001	0.0036	1.06E-06	4.52E-03	Yes
cg07553761	0.048 ± 0.001	0.053 ± 0.001	0.054 ± 0.001	0.0026	9.66E-06	2.86E-02	Yes
cg04098479	0.958 ± 0	0.961 ± 0.001	0.961 ± 0.001	0.0021	5.76E-07	2.89E-03	Yes
cg16449012	0.718 ± 0.002	0.728 ± 0.004	0.73 ± 0.003	0.0061	5.64E-05	9.13E-02	No
cg14349977	0.219 ± 0.003	0.199 ± 0.006	0.197 ± 0.006	-0.0133	1.44E-05	3.73E-02	Yes
cg18753646	0.165 ± 0.003	0.141 ± 0.006	0.149 ± 0.005	-0.0111	4.82E-05	8.67E-02	No
cg17924476	0.326 ± 0.002	0.336 ± 0.004	0.357 ± 0.004	0.0121	1.67E-08	1.28E-04	Yes
cg23067299	0.82 ± 0.002	0.838 ± 0.004	0.846 ± 0.004	0.0139	4.39E-13	6.47E-09	Yes
cg08606254	0.857 ± 0.001	0.873 ± 0.003	0.882 ± 0.003	0.0121	2.13E-15	3.74E-11	Yes
cg12806681	0.918 ± 0.001	0.906 ± 0.003	0.913 ± 0.002	-0.0047	3.05E-06	1.11E-02	No
cg03991871	0.891 ± 0.002	0.882 ± 0.003	0.885 ± 0.003	-0.0052	5.71E-06	1.87E-02	No
cg23916896	0.186 ± 0.002	0.165 ± 0.004	0.163 ± 0.004	-0.0127	6.47E-08	4.50E-04	Yes
cg11902777	0.049 ± 0.001	0.039 ± 0.001	0.042 ± 0.002	-0.0045	2.97E-09	2.73E-05	No
cg05575921	0.891 ± 0.002	0.855 ± 0.005	0.84 ± 0.005	-0.0294	3.55E-74	1.31E-68	Yes
cg14817490	0.313 ± 0.002	0.288 ± 0.005	0.296 ± 0.005	-0.0122	1.35E-07	8.91E-04	No

Probe	Non-active[b]	Light[b]	Moderate / Heavy[b]	Coeff[c]	*P*-value[c]	*q*-value[c]	Dose-response[d]
cg21161138	0.822 ± 0.001	0.804 ± 0.002	0.8 ± 0.003	-0.0113	4.94E-24	1.65E-19	Yes
cg22937882	0.903 ± 0.001	0.915 ± 0.001	0.916 ± 0.002	0.0079	1.35E-19	3.55E-15	Yes
cg07448928	0.847 ± 0.001	0.853 ± 0.002	0.854 ± 0.002	0.0044	3.55E-05	7.47E-02	No
cg10841124	0.943 ± 0.001	0.948 ± 0.001	0.949 ± 0.001	0.0025	4.26E-06	1.47E-02	Yes
cg26954197	0.914 ± 0.001	0.921 ± 0.002	0.92 ± 0.001	0.0038	7.28E-06	2.23E-02	No
cg22879098	0.443 ± 0.004	0.411 ± 0.008	0.423 ± 0.009	-0.0158	2.12E-04	1.97E-01	No
cg01952185	0.636 ± 0.003	0.659 ± 0.006	0.675 ± 0.006	0.0185	5.23E-13	7.13E-09	Yes
cg11429111	0.766 ± 0.003	0.793 ± 0.006	0.807 ± 0.006	0.0194	4.80E-13	6.80E-09	Yes
cg25383593	0.129 ± 0.002	0.139 ± 0.003	0.144 ± 0.003	0.0072	2.48E-06	9.53E-03	Yes
cg25415695	0.602 ± 0.002	0.611 ± 0.004	0.616 ± 0.004	0.0076	1.37E-05	3.66E-02	Yes
cg04800195	0.042 ± 0	0.046 ± 0.001	0.045 ± 0.001	0.0019	2.29E-05	5.40E-02	No
cg26889659	0.314 ± 0.007	0.279 ± 0.013	0.256 ± 0.012	-0.0346	5.91E-09	4.94E-05	Yes
cg18694169	0.159 ± 0.002	0.181 ± 0.005	0.176 ± 0.004	0.0101	4.76E-06	1.61E-02	No
cg10253847	0.071 ± 0.001	0.088 ± 0.003	0.085 ± 0.003	0.007	1.04E-06	4.50E-03	No
cg01031101	0.253 ± 0.002	0.278 ± 0.005	0.281 ± 0.005	0.0127	3.22E-08	2.32E-04	Yes
cg09523275	0.317 ± 0.002	0.341 ± 0.005	0.338 ± 0.004	0.009	5.10E-05	8.95E-02	No
cg18675097	0.486 ± 0.003	0.524 ± 0.007	0.513 ± 0.007	0.013	2.87E-04	2.27E-01	No
cg18828927	0.772 ± 0.001	0.779 ± 0.002	0.778 ± 0.002	0.0047	5.97E-05	9.40E-02	No
cg14027333	0.056 ± 0.001	0.052 ± 0.001	0.052 ± 0.001	-0.0027	5.42E-05	9.06E-02	No
cg02404974	0.772 ± 0.002	0.789 ± 0.004	0.785 ± 0.004	0.0078	2.19E-04	1.99E-01	No
cg14086013	0.746 ± 0.002	0.76 ± 0.003	0.761 ± 0.004	0.0072	2.20E-05	5.29E-02	No
cg03354508	0.869 ± 0.001	0.878 ± 0.003	0.881 ± 0.003	0.0068	8.81E-06	2.64E-02	Yes
cg17345450	0.792 ± 0.002	0.782 ± 0.003	0.775 ± 0.003	-0.0068	8.04E-06	2.45E-02	Yes
cg00794911	0.889 ± 0.001	0.88 ± 0.003	0.876 ± 0.002	-0.0063	5.81E-07	2.89E-03	Yes
cg18132363	0.453 ± 0.002	0.428 ± 0.005	0.421 ± 0.005	-0.0166	1.09E-10	1.18E-06	Yes
cg11625488	0.461 ± 0.003	0.439 ± 0.008	0.445 ± 0.008	-0.0124	3.49E-05	7.42E-02	No
cg10312186	0.377 ± 0.003	0.349 ± 0.005	0.354 ± 0.005	-0.0145	4.45E-07	2.40E-03	No

Probe	Non-active[b]	Light[b]	Moderate / Heavy[b]	Coeff[c]	*P*-value[c]	*q*-value[c]	Dose-response[d]
cg16944958	0.814 ± 0.002	0.805 ± 0.004	0.804 ± 0.004	-0.0072	4.72E-05	8.67E-02	No
cg07249149	0.673 ± 0.002	0.657 ± 0.004	0.658 ± 0.004	-0.0075	5.16E-05	8.98E-02	No
cg07104557	0.718 ± 0.002	0.704 ± 0.003	0.698 ± 0.003	-0.0084	1.75E-07	1.10E-03	Yes
cg19089201	0.923 ± 0.002	0.942 ± 0.003	0.945 ± 0.003	0.0093	3.94E-20	1.12E-15	Yes
cg22132788	0.953 ± 0.001	0.967 ± 0.002	0.969 ± 0.002	0.0084	5.01E-17	1.03E-12	Yes
cg04180046	0.531 ± 0.002	0.578 ± 0.004	0.587 ± 0.005	0.0313	2.12E-40	1.95E-35	Yes
cg12803068	0.844 ± 0.003	0.893 ± 0.005	0.9 ± 0.005	0.0311	3.03E-28	1.40E-23	Yes
cg16459265	0.832 ± 0.001	0.824 ± 0.002	0.818 ± 0.002	-0.0061	2.13E-07	1.25E-03	Yes
cg04598670	0.609 ± 0.003	0.601 ± 0.007	0.585 ± 0.006	-0.0144	6.07E-09	4.97E-05	Yes
cg18209323	0.418 ± 0.002	0.436 ± 0.005	0.433 ± 0.005	0.0085	2.01E-04	1.93E-01	No
cg20174893	0.194 ± 0.002	0.183 ± 0.004	0.187 ± 0.004	-0.0061	5.17E-05	8.98E-02	No
cg07464358	0.384 ± 0.002	0.392 ± 0.003	0.397 ± 0.003	0.0046	2.75E-05	6.40E-02	No
cg25949550	0.116 ± 0.001	0.096 ± 0.003	0.098 ± 0.002	-0.0111	6.93E-27	2.84E-22	No
cg15578140	0.671 ± 0.001	0.686 ± 0.003	0.684 ± 0.003	0.0076	7.00E-07	3.35E-03	No
cg00433296	0.743 ± 0.002	0.753 ± 0.003	0.753 ± 0.004	0.0057	5.22E-05	8.98E-02	No
cg13455434	0.554 ± 0.004	0.573 ± 0.006	0.589 ± 0.008	0.0175	2.82E-06	1.06E-02	Yes
cg02892925	0.604 ± 0.002	0.627 ± 0.005	0.618 ± 0.005	0.0105	3.62E-05	7.49E-02	No
cg07211044	0.394 ± 0.003	0.423 ± 0.006	0.416 ± 0.007	0.0122	3.31E-05	7.22E-02	No
cg18794473	0.941 ± 0.001	0.944 ± 0.001	0.944 ± 0.001	0.0024	3.73E-05	7.55E-02	No
cg04640109	0.234 ± 0.002	0.242 ± 0.003	0.253 ± 0.003	0.0071	1.03E-06	4.50E-03	Yes
cg11064524	0.795 ± 0.002	0.786 ± 0.003	0.779 ± 0.003	-0.0069	1.56E-05	4.03E-02	Yes
cg13459104	0.961 ± 0	0.965 ± 0.001	0.965 ± 0.001	0.0018	5.67E-05	9.13E-02	No
cg01825213	0.667 ± 0.003	0.682 ± 0.005	0.689 ± 0.006	0.0123	1.90E-06	7.71E-03	Yes
cg13784312	0.239 ± 0.003	0.219 ± 0.005	0.218 ± 0.005	-0.0091	4.83E-05	8.67E-02	No
cg13822849	0.126 ± 0.001	0.138 ± 0.004	0.145 ± 0.003	0.0093	5.28E-09	4.52E-05	Yes
cg20344448	0.772 ± 0.002	0.785 ± 0.003	0.789 ± 0.003	0.0089	5.11E-09	4.48E-05	Yes
cg11813497	0.773 ± 0.003	0.794 ± 0.006	0.809 ± 0.005	0.0148	3.87E-09	3.48E-05	Yes

Probe	Non-active[b]	Light[b]	Moderate / Heavy[b]	Coeff[c]	*P*-value[c]	*q*-value[c]	Dose-response[d]
cg25464840	0.688 ± 0.002	0.71 ± 0.004	0.713 ± 0.003	0.0126	1.17E-13	1.88E-09	Yes
cg15507334	0.559 ± 0.002	0.579 ± 0.004	0.583 ± 0.003	0.0114	3.02E-12	3.59E-08	Yes
cg03486379	0.781 ± 0.001	0.791 ± 0.003	0.789 ± 0.003	0.0055	4.12E-05	7.86E-02	No
cg24239148	0.037 ± 0.001	0.044 ± 0.002	0.045 ± 0.002	0.0041	2.90E-05	6.64E-02	No
cg06813382	0.702 ± 0.002	0.725 ± 0.004	0.718 ± 0.005	0.0093	6.20E-05	9.55E-02	No
cg08626436	0.486 ± 0.004	0.511 ± 0.008	0.538 ± 0.008	0.0221	7.68E-08	5.24E-04	Yes
cg09488203	0.158 ± 0.001	0.166 ± 0.003	0.167 ± 0.002	0.0053	1.34E-05	3.61E-02	Yes
cg05593775	0.492 ± 0.002	0.507 ± 0.005	0.521 ± 0.005	0.0108	8.45E-07	3.89E-03	Yes
cg18621256	0.526 ± 0.002	0.507 ± 0.003	0.514 ± 0.004	-0.0071	2.87E-05	6.60E-02	No
cg10673740	0.351 ± 0.002	0.362 ± 0.005	0.381 ± 0.005	0.013	2.04E-07	1.23E-03	Yes
cg22043296	0.769 ± 0.002	0.752 ± 0.004	0.755 ± 0.004	-0.0059	2.54E-04	2.14E-01	No
cg19631472	0.477 ± 0.001	0.467 ± 0.003	0.464 ± 0.003	-0.0072	9.99E-07	4.43E-03	Yes
cg10788371	0.384 ± 0.001	0.374 ± 0.003	0.376 ± 0.003	-0.0054	1.59E-04	1.69E-01	No
cg09063262	0.311 ± 0.003	0.299 ± 0.006	0.282 ± 0.005	-0.0151	7.83E-07	3.65E-03	Yes
cg19563365	0.887 ± 0.001	0.892 ± 0.002	0.895 ± 0.002	0.0044	1.18E-05	3.30E-02	Yes
cg02356218	0.543 ± 0.002	0.531 ± 0.004	0.529 ± 0.004	-0.0081	1.90E-05	4.67E-02	Yes
cg26827653	0.719 ± 0.002	0.727 ± 0.003	0.736 ± 0.004	0.0086	1.44E-06	6.05E-03	Yes
cg10520740	0.805 ± 0.001	0.8 ± 0.002	0.79 ± 0.002	-0.0065	1.33E-12	1.63E-08	Yes
cg22171758	0.792 ± 0.002	0.801 ± 0.004	0.803 ± 0.004	0.0071	3.40E-05	7.33E-02	No
cg25122233	0.526 ± 0.003	0.548 ± 0.006	0.541 ± 0.006	0.0102	3.47E-04	2.51E-01	No
cg03453449	0.749 ± 0.002	0.766 ± 0.004	0.765 ± 0.004	0.0102	7.71E-07	3.64E-03	No
cg25632883	0.381 ± 0.004	0.343 ± 0.008	0.348 ± 0.009	-0.02	6.52E-06	2.07E-02	No
cg24698536	0.311 ± 0.003	0.288 ± 0.007	0.289 ± 0.008	-0.0147	1.67E-05	4.18E-02	No
cg12492906	0.444 ± 0.003	0.421 ± 0.006	0.427 ± 0.006	-0.0115	3.18E-05	7.11E-02	No
cg00029284	0.79 ± 0.001	0.786 ± 0.002	0.778 ± 0.003	-0.006	5.98E-07	2.94E-03	Yes
cg26103179	0.177 ± 0.002	0.16 ± 0.004	0.169 ± 0.004	-0.0076	2.79E-04	2.22E-01	No
cg12160087	0.489 ± 0.001	0.482 ± 0.003	0.477 ± 0.003	-0.0064	3.98E-06	1.38E-02	Yes

Probe	Non-active[b]	Light[b]	Moderate / Heavy[b]	Coeff[c]	*P*-value[c]	*q*-value[c]	Dose-response[d]
cg15893360	0.635 ± 0.002	0.621 ± 0.004	0.625 ± 0.004	-0.0065	4.74E-04	2.93E-01	No
cg08698721	0.658 ± 0.002	0.666 ± 0.004	0.673 ± 0.004	0.0084	2.20E-06	8.71E-03	Yes
cg04291079	0.897 ± 0.002	0.909 ± 0.003	0.911 ± 0.004	0.0077	5.62E-07	2.88E-03	Yes
cg26554302	0.597 ± 0.003	0.614 ± 0.005	0.62 ± 0.005	0.0083	1.13E-05	3.19E-02	Yes
cg03796381	0.67 ± 0.001	0.682 ± 0.003	0.687 ± 0.003	0.0096	5.82E-10	5.80E-06	Yes
cg10078511	0.814 ± 0.002	0.83 ± 0.003	0.824 ± 0.003	0.0068	5.71E-05	9.13E-02	No
cg23849826	0.9 ± 0.001	0.912 ± 0.002	0.909 ± 0.002	0.0047	8.13E-05	1.14E-01	No
cg16231923	0.596 ± 0.003	0.616 ± 0.006	0.622 ± 0.006	0.0121	1.23E-05	3.37E-02	Yes
cg00213123	0.154 ± 0.001	0.166 ± 0.003	0.17 ± 0.003	0.0074	3.42E-10	3.60E-06	Yes
cg05549655	0.198 ± 0.002	0.228 ± 0.006	0.239 ± 0.005	0.0191	3.52E-17	7.63E-13	Yes
cg17852385	0.301 ± 0.003	0.321 ± 0.007	0.334 ± 0.006	0.0143	6.75E-07	3.27E-03	Yes
cg13570656	0.308 ± 0.003	0.344 ± 0.008	0.359 ± 0.008	0.026	6.69E-13	8.81E-09	Yes
cg12101586	0.398 ± 0.003	0.437 ± 0.007	0.445 ± 0.007	0.0235	5.00E-14	8.37E-10	Yes
cg22549041	0.345 ± 0.005	0.404 ± 0.01	0.418 ± 0.01	0.0351	2.40E-16	4.66E-12	Yes
cg11924019	0.474 ± 0.002	0.5 ± 0.005	0.513 ± 0.005	0.0178	3.10E-18	7.13E-14	Yes
cg18092474	0.562 ± 0.004	0.598 ± 0.008	0.624 ± 0.008	0.0264	2.33E-13	3.58E-09	Yes
cg26516004	0.94 ± 0.001	0.949 ± 0.002	0.95 ± 0.002	0.0047	2.48E-06	9.53E-03	Yes
cg01359532	0.854 ± 0.001	0.865 ± 0.003	0.872 ± 0.003	0.0089	2.89E-09	2.73E-05	Yes
cg04807108	0.724 ± 0.003	0.752 ± 0.006	0.749 ± 0.006	0.0123	9.17E-05	1.22E-01	No
cg12937151	0.509 ± 0.004	0.489 ± 0.007	0.477 ± 0.007	-0.0173	2.10E-06	8.42E-03	Yes
cg04459939	0.485 ± 0.003	0.51 ± 0.006	0.519 ± 0.007	0.0145	1.11E-07	7.42E-04	Yes
cg23335299	0.453 ± 0.003	0.48 ± 0.006	0.487 ± 0.007	0.0144	4.70E-07	2.47E-03	Yes
cg13834112	0.653 ± 0.003	0.677 ± 0.005	0.688 ± 0.005	0.014	2.60E-08	1.91E-04	Yes
cg26804595	0.458 ± 0.002	0.443 ± 0.003	0.457 ± 0.003	-0.0044	1.88E-03	4.87E-01	No
cg13928411	0.617 ± 0.001	0.626 ± 0.003	0.623 ± 0.003	0.0049	8.59E-05	1.18E-01	No
cg09743950	0.759 ± 0.002	0.744 ± 0.004	0.741 ± 0.004	-0.0091	6.90E-06	2.15E-02	Yes
cg00253658	0.319 ± 0.004	0.375 ± 0.009	0.389 ± 0.009	0.0343	5.58E-16	1.03E-11	Yes

Probe	Non-active[b]	Light[b]	Moderate / Heavy[b]	Coeff[c]	*P*-value[c]	*q*-value[c]	Dose-response[d]
cg26681628	0.777 ± 0.002	0.801 ± 0.003	0.799 ± 0.003	0.0118	6.52E-10	6.32E-06	No
cg03687532	0.904 ± 0.002	0.921 ± 0.003	0.915 ± 0.003	0.0057	7.84E-04	3.54E-01	No
cg03177666	0.763 ± 0.002	0.78 ± 0.004	0.785 ± 0.004	0.0097	2.92E-06	1.08E-02	Yes
cg08755703	0.612 ± 0.002	0.627 ± 0.005	0.641 ± 0.005	0.0116	2.71E-06	1.03E-02	Yes
cg24173182	0.335 ± 0.003	0.354 ± 0.006	0.364 ± 0.005	0.0135	6.81E-06	2.15E-02	Yes
cg05568941	0.764 ± 0.002	0.784 ± 0.005	0.782 ± 0.004	0.0082	7.02E-06	2.17E-02	No
cg06949812	0.486 ± 0.002	0.502 ± 0.005	0.5 ± 0.004	0.0066	2.59E-04	2.16E-01	No
cg11096905	0.671 ± 0.002	0.653 ± 0.004	0.659 ± 0.004	-0.0082	8.87E-05	1.20E-01	No
cg23928512	0.569 ± 0.002	0.558 ± 0.004	0.554 ± 0.004	-0.0066	1.58E-05	4.04E-02	Yes
cg23664708	0.253 ± 0.002	0.244 ± 0.005	0.236 ± 0.005	-0.0113	3.33E-07	1.91E-03	Yes
cg06509837	0.341 ± 0.003	0.313 ± 0.007	0.319 ± 0.007	-0.0136	2.62E-04	2.17E-01	No
cg26580869	0.752 ± 0.002	0.726 ± 0.006	0.738 ± 0.005	-0.0091	1.09E-03	4.00E-01	No
cg02010738	0.43 ± 0.003	0.402 ± 0.006	0.419 ± 0.006	-0.0087	1.35E-03	4.39E-01	No
cg08097581	0.604 ± 0.001	0.591 ± 0.003	0.593 ± 0.002	-0.0058	1.85E-06	7.58E-03	No
cg26652413	0.108 ± 0.002	0.119 ± 0.003	0.123 ± 0.003	0.0067	1.60E-06	6.63E-03	Yes
cg23458168	0.253 ± 0.004	0.275 ± 0.008	0.297 ± 0.009	0.0186	4.93E-06	1.65E-02	Yes
cg16547579	0.726 ± 0.002	0.708 ± 0.004	0.718 ± 0.004	-0.0075	3.73E-04	2.59E-01	No
cg20244340	0.353 ± 0.002	0.336 ± 0.004	0.339 ± 0.004	-0.0099	3.99E-07	2.23E-03	No
cg11868728	0.848 ± 0.001	0.857 ± 0.003	0.855 ± 0.003	0.0049	3.11E-05	7.03E-02	No
cg20274009	0.781 ± 0.001	0.787 ± 0.003	0.784 ± 0.003	0.0038	3.83E-05	7.59E-02	No
cg07339236	0.081 ± 0.001	0.071 ± 0.002	0.066 ± 0.002	-0.008	6.00E-19	1.47E-14	Yes
cg20643029	0.188 ± 0.001	0.178 ± 0.003	0.173 ± 0.003	-0.0074	3.07E-06	1.11E-02	Yes
cg12426652	0.123 ± 0.001	0.117 ± 0.003	0.115 ± 0.002	-0.0051	5.50E-06	1.82E-02	Yes
cg04478905	0.442 ± 0.002	0.43 ± 0.004	0.428 ± 0.003	-0.009	8.95E-07	4.02E-03	Yes
cg26441486	0.415 ± 0.002	0.4 ± 0.005	0.392 ± 0.005	-0.0116	2.28E-06	8.92E-03	Yes

[a]FDR q<0.05 (Model1). [b]β ± SE for the ratio of methylated signal to total signal (methylated plus unmethylated). [c]3-level maternal smoking variable (non-active, light, moderate/heavy) treated as a continuous variable. [d]Notes whether or not the CpG site meets our criteria for showing a dose-response (see Methods section, Tobacco smoke exposure, for details).

Table S7. Percentage of CpGs showing a dose-response relationship.

P-value cutoff	N CpG sites	% Dose-response
$p < 1x10^{-4}$	185[a]	57.8%
$p < 1x10^{-5}$	140	65.0%
$p < 1x10^{-6}$	87	75.9%
$p < 1x10^{-7}$	63	79.4%
$p < 1x10^{-8}$	47	87.2%
$p < 1x10^{-9}$	39	89.7%
$p < 1x10^{-10}$	34	91.2%

[a]All sites had an FDR $q < 0.05$.

Table S8. Genome-wide significant CpG sites: replication using previously published studies.[a]

Probe	Chr	Basepair[b]	Nearest gene	Newborn[c]: Site	Newborn[c]: Gene	Adult[c]: Site	Adult[c]: Gene	Model1[d] Coeff	Model1[d] *P*-value	Model1[d] *q*-value	Δ β-value[e]
cg05575921	5	373378	*AHRR*	J	J	Z; S	Z; S; M	-0.046	1.45E-69[*]	5.17E-64	-0.043
cg04180046	7	45002736	*MYO1G*	J	J	Z	Z	0.051	1.53E-40[*]	2.73E-35	0.052
cg09935388	1	92947588	*GFI1*	J	J	Z; S	Z; S	-0.086	2.39E-34[*]	2.85E-29	-0.084
cg25949550	7	145814306	*CNTNAP2*	J	J	Z; S	Z; S	-0.020	1.39E-31[*]	1.24E-26	-0.019
cg12876356	1	92946825	*GFI1*	J	J	Z	Z; S	-0.069	2.22E-31[*]	1.59E-26	-0.077
cg12803068	7	45002919	*MYO1G*	J	J	Z	Z	0.053	3.58E-31[*]	2.13E-26	0.053
cg18146737	1	92946700	*GFI1*	J	J	Z	Z; S	-0.065	3.83E-30[*]	1.96E-25	-0.075
cg18316974	1	92947035	*GFI1*	J	J	Z	Z; S	-0.033	3.47E-29[*]	1.55E-24	-0.044
cg21161138	5	399360	*AHRR*	J	J	Z; S	Z; S; M	-0.019	5.51E-26[*]	2.19E-21	-0.020
cg19089201	7	45002287	*MYO1G*	J	J	Z	Z	0.017	1.62E-23[*]	5.79E-19	0.020
cg14179389	1	92947961	*GFI1*	J	J	NA	Z; S	-0.068	3.38E-23[*]	1.10E-18	-0.065
cg06338710	1	92946187	*GFI1*	J	J	NA	Z; S	-0.023	1.96E-21[*]	5.83E-17	-0.031
cg22937882	5	405774	*AHRR*	NA	J	NA	Z; S; M	0.013	2.76E-21[*]	7.58E-17	0.013
cg09662411	1	92946132	*GFI1*	J	J	Z	Z; S	-0.034	1.06E-19[*]	2.69E-15	-0.043
cg22132788	7	45002486	*MYO1G*	J	J	Z	Z	0.015	2.11E-19[*]	5.04E-15	0.016
cg07339236	20	50312490	*ATP9A*	J	J	Z	Z	-0.013	2.21E-18[*]	4.94E-14	-0.013
cg05549655	15	75019143	*CYP1A1*	J	J	NA	NA	0.032	7.92E-18[*]	1.66E-13	0.036
cg11924019	15	75019283	*CYP1A1*	J	J	NA	NA	0.029	9.40E-18[*]	1.87E-13	0.033
cg00253658	16	54210496	*FTO*	J	J	NA	NA	0.059	1.37E-17[*]	2.57E-13	0.063
cg22549041	15	75019251	*CYP1A1*	J	J	NA	NA	0.059	5.94E-17[*]	1.06E-12	0.066
cg08606254	5	323969	*AHRR*	NA	J	Z	Z; S; M	0.020	2.14E-15[*]	3.65E-11	0.021
cg25464840	10	14372910	*FRMD4A*	NA	J	NA	NA	0.022	3.15E-15[*]	5.11E-11	0.023
cg12101586	15	75019203	*CYP1A1*	J	J	NA	NA	0.039	5.38E-15[*]	8.36E-11	0.043
cg15507334	10	14372913	*FRMD4A*	NA	J	NA	NA	0.020	2.43E-13[*]	3.62E-09	0.022
cg26681628	16	54210550	*FTO*	NA	J	NA	NA	0.023	4.05E-13[*]	5.79E-09	0.023
cg18092474	15	75019302	*CYP1A1*	J	J	NA	NA	0.042	5.47E-13[*]	7.51E-09	0.050
cg23067299	5	323907	*AHRR*	J	J	NA	Z; S; M	0.022	8.94E-13[*]	1.18E-08	0.022
cg13570656	15	75019196	*CYP1A1*	J	J	NA	NA	0.042	9.63E-13[*]	1.23E-08	0.044
cg11429111	5	134813329	*TIFAB*	NA	NA	NA	NA	0.031	1.04E-12[*]	1.29E-08	0.034
cg01952185	5	134813213	*TIFAB*	NA	NA	NA	NA	0.029	2.55E-12[*]	3.04E-08	0.031
cg18703066	2	105363536	*LOC284998*	J	J	NA	NA	-0.006	7.63E-12[*]	8.79E-08	-0.004

Probe	Chr	Basepair[b]	Nearest gene	Newborn[c]: Site	Newborn[c]: Gene	Adult[c]: Site	Adult[c]: Gene	Model1[d] Coeff	Model1[d] *P*-value	Model1[d] *q*-value	Δ β-value[e]
cg11902777	5	368843	*AHRR*	NA	J	Z	Z; S; M	-0.008	1.13E-11*	1.26E-07	-0.009
cg18132363	6	166260572	*SDIM1*	NA	NA	NA	NA	-0.027	8.92E-11*	9.53E-07	-0.029
cg15325070	1	2792704	*TTC34*	NA	NA	NA	NA	0.018	9.07E-11*	9.53E-07	0.018
cg10399789	1	92945668	*GFI1*	J	J	NA	Z; S	-0.016	5.74E-10*	5.66E-06	-0.020
cg14817490	5	392920	*AHRR*	NA	J	Z	Z; S; M	-0.023	5.75E-10*	5.66E-06	-0.021
cg01031101	6	28227091	*NKAPL*	NA	NA	NA	NA	0.023	5.86E-10*	5.66E-06	0.026
cg26764244	1	68299511	*GNG12*	J	J	NA	Z; S	-0.023	6.68E-10*	6.28E-06	-0.023
cg00213123	15	75019070	*CYP1A1*	NA	J	NA	NA	0.012	7.47E-10*	6.85E-06	0.014
cg03796381	15	36650033	*COX6CP4*	NA	NA	NA	NA	0.015	1.17E-09*	1.05E-05	0.015
cg10520740	12	1952798	*CACNA2D4*	NA	NA	NA	NA	-0.009	1.86E-09*	1.62E-05	-0.010
cg03144619	1	230415668	*GALNT2*	NA	J	NA	NA	-0.037	2.17E-09*	1.85E-05	-0.038
cg20344448	10	14372431	*FRMD4A*	NA	J	NA	NA	0.015	2.97E-09*	2.47E-05	0.015
cg01359532	15	75045126	*CYP1A2*	NA	NA	NA	NA	0.014	3.11E-09*	2.53E-05	0.015
cg10253847	6	28227085	*NKAPL*	NA	NA	NA	NA	0.013	6.51E-09*	5.17E-05	0.015
cg11813497	10	14372879	*FRMD4A*	J	J	NA	NA	0.024	6.85E-09*	5.32E-05	0.029
cg25189904	1	68299493	*GNG12*	J	J	Z; S	Z; S	-0.020	9.06E-09*	6.89E-05	-0.018
cg24250902	1	230415547	*GALNT2*	NA	J	NA	NA	-0.032	1.13E-08*	8.43E-05	-0.031
cg13822849	9	137999757	*OLFM1*	NA	NA	NA	NA	0.015	1.18E-08*	8.58E-05	0.015
cg23916896	5	368804	*AHRR*	J	J	Z	Z; S; M	-0.022	1.24E-08*	8.87E-05	-0.022
cg13834112	15	90361639	*ANPEP*	NA	NA	NA	Z	0.023	1.46E-08*	1.00E-04	0.029
cg26889659	6	684090	*EXOC2*	NA	NA	NA	NA	-0.055	1.46E-08*	1.00E-04	-0.047
cg12806681	5	368394	*AHRR*	NA	J	Z	Z; S; M	-0.009	1.59E-08*	1.07E-04	-0.008
cg05697274	1	230415377	*GALNT2*	NA	J	NA	NA	-0.026	1.75E-08*	1.16E-04	-0.024
cg18096987	3	11623873	*VGLL4*	J	J	NA	NA	-0.018	4.10E-08*	2.67E-04	-0.013
cg10312186	6	170403583	*LOC100505903*	NA	NA	NA	NA	-0.026	4.40E-08*	2.81E-04	-0.025
cg08097581	19	15528589	*AKAP8L*	NA	NA	NA	NA	-0.011	5.15E-08*	3.21E-04	-0.012
cg07104557	7	1036653	*C7orf50*	NA	NA	NA	NA	-0.014	5.21E-08*	3.21E-04	-0.017
cg15578140	7	147718109	*CNTNAP2*	NA	J	NA	Z; S	0.014	5.56E-08*	3.32E-04	0.015
cg23335299	15	90291756	*MESP1*	NA	NA	NA	NA	0.025	5.58E-08*	3.32E-04	0.030
cg20244340	20	19193989	*SLC24A3*	NA	NA	NA	Z	-0.017	6.33E-08*	3.71E-04	-0.015
cg04459939	15	90291141	*MESP1*	NA	NA	NA	NA	0.024	6.91E-08*	3.98E-04	0.029
cg18694169	6	28227079	*NKAPL*	NA	NA	NA	NA	0.019	9.62E-08*	5.46E-04	0.020
cg00589617	1	230415343	*GALNT2*	NA	J	NA	NA	-0.029	1.07E-07*	5.89E-04	-0.029
cg04598670	7	68697651	*LOC100507468*	J	J	NA	NA	-0.021	1.07E-07*	5.89E-04	-0.016
cg24591105	1	230415225	*GALNT2*	NA	J	NA	NA	-0.024	1.26E-07*	6.83E-04	-0.026

Probe	Chr	Basepair[b]	Nearest gene	Newborn[c]: Site	Newborn[c]: Gene	Adult[c]: Site	Adult[c]: Gene	Model1[d] Coeff	Model1[d] *P*-value	Model1[d] *q*-value	Δ β-value[e]
cg04098479	4	1994849	*WHSC2*	NA	NA	NA	NA	0.004	1.29E-07*	6.89E-04	0.003
cg03453449	12	95945120	*USP44*	NA	NA	NA	NA	0.018	1.31E-07*	6.89E-04	0.017
cg09368188	1	245330018	*KIF26B*	NA	J	NA	NA	0.026	1.54E-07	7.95E-04	0.030
cg04291079	14	101294430	*MEG3*	NA	J	NA	NA	0.013	1.67E-07	8.53E-04	0.013
cg05568941	17	38517913	*GJD3*	NA	NA	NA	NA	0.015	1.78E-07	8.94E-04	0.019
cg04478905	21	37915091	*CLDN14*	NA	NA	NA	NA	-0.016	1.96E-07	9.70E-04	-0.013
cg15522425	1	2792637	*TTC34*	NA	NA	NA	NA	0.014	2.36E-07	1.16E-03	0.014
cg18621256	10	119221444	*EMX2OS*	NA	NA	NA	NA	-0.014	3.95E-07	1.91E-03	-0.015
cg26954197	5	436816	*AHRR*	NA	J	NA	Z; S; M	0.007	4.80E-07	2.29E-03	0.006
cg03991871	5	368447	*AHRR*	J	J	Z	Z; S; M	-0.009	5.01E-07	2.36E-03	-0.008
cg19631472	11	18415641	*LDHA*	NA	NA	NA	NA	-0.012	5.28E-07	2.45E-03	-0.011
cg16459265	7	45025080	*C7orf40*	NA	NA	NA	Z	-0.010	6.22E-07	2.85E-03	-0.011
cg26119806	2	18061572	*KCNS3*	NA	NA	NA	NA	-0.017	6.56E-07	2.97E-03	-0.017
cg03177666	16	54365103	*IRX3*	NA	NA	NA	NA	0.017	7.08E-07	3.16E-03	0.019
cg17852385	15	75019188	*CYP1A1*	NA	J	NA	NA	0.023	7.21E-07	3.18E-03	0.026
cg25632883	12	109250438	*SSH1*	NA	NA	NA	NA	-0.036	7.61E-07	3.31E-03	-0.035
cg20643029	21	37915044	*CLDN14*	NA	NA	NA	NA	-0.013	7.70E-07	3.31E-03	-0.013
cg00794911	6	166260532	*SDIM1*	NA	NA	NA	NA	-0.010	8.10E-07	3.45E-03	-0.011
cg02892925	8	60032926	*LOC100505501*	NA	NA	NA	NA	0.020	8.48E-07	3.56E-03	0.018
cg09523275	6	28227093	*NKAPL*	NA	NA	NA	NA	0.018	8.79E-07	3.65E-03	0.022
cg17924476	5	323794	*AHRR*	NA	J	Z	Z; S; M	0.017	9.38E-07	3.85E-03	0.020
cg15836046	1	2767242	*TTC34*	NA	NA	NA	NA	0.006	1.03E-06	4.19E-03	0.005
cg12426652	21	37915056	*CLDN14*	NA	NA	NA	NA	-0.009	1.18E-06	4.73E-03	-0.007
cg26516004	15	75019376	*CYP1A1*	NA	J	NA	NA	0.008	1.20E-06	4.77E-03	0.009
cg08626436	10	88555339	*BMPR1A*	NA	NA	NA	NA	0.032	1.57E-06	6.15E-03	0.038
cg18753646	4	187219538	*LOC285441*	NA	NA	NA	NA	-0.022	1.60E-06	6.22E-03	-0.019
cg07211044	8	60032983	*LOC100505501*	NA	NA	NA	NA	0.023	1.65E-06	6.32E-03	0.025
cg23664708	17	80196903	*SLC16A3*	NA	NA	NA	NA	-0.017	1.70E-06	6.35E-03	-0.013
cg09743950	16	31341875	*ITGAM*	NA	NA	NA	NA	-0.016	1.71E-06	6.35E-03	-0.016
cg18675097	6	28227127	*NKAPL*	NA	NA	NA	NA	0.028	1.71E-06	6.35E-03	0.032
cg26652413	19	17007057	*CPAMD8*	NA	NA	NA	Z	0.011	1.94E-06	7.16E-03	0.013
cg18722086	3	113380072	*KIAA2018*	NA	NA	NA	NA	0.006	2.40E-06	8.68E-03	0.005
cg01825213	9	98979965	*HSD17B3*	NA	NA	NA	NA	0.020	2.41E-06	8.68E-03	0.019
cg25383593	5	134827820	*TIFAB*	NA	NA	NA	NA	0.012	2.56E-06	9.09E-03	0.013
cg20816361	1	2467100	*HES5*	NA	NA	NA	NA	0.016	2.60E-06	9.09E-03	0.015

Probe	Chr	Basepair[b]	Nearest gene	Newborn[c]: Site	Newborn[c]: Gene	Adult[c]: Site	Adult[c]: Gene	Model1[d] Coeff	Model1[d] *P*-value	Model1[d] *q*-value	Δ β-value[e]
cg10078511	15	39872032	*THBS1*	NA	NA	NA	NA	0.013	2.61E-06	9.09E-03	0.013
cg06813382	10	81967195	*LOC439990*	NA	NA	NA	NA	0.018	2.62E-06	9.09E-03	0.019
cg06415891	1	2467096	*HES5*	NA	NA	NA	NA	0.019	2.74E-06	9.41E-03	0.018
cg17442776	1	147718540	*TRNAQ8*	NA	NA	NA	NA	0.009	3.14E-06	1.06E-02	0.008
cg23849826	15	39872186	*THBS1*	NA	NA	NA	NA	0.009	3.16E-06	1.06E-02	0.010
cg08026735	2	98703888	*VWA3B*	NA	NA	NA	NA	0.012	3.16E-06	1.06E-02	0.012
cg11625488	6	170403557	*LOC100505903*	NA	NA	NA	NA	-0.022	3.70E-06	1.22E-02	-0.019
cg10841124	5	433274	*AHRR*	NA	J	NA	Z; S; M	0.004	3.79E-06	1.24E-02	0.005
cg12937151	15	87758226	*AGBL1*	NA	NA	NA	NA	-0.028	3.89E-06	1.26E-02	-0.026
cg16547579	20	4954333	*SLC23A2*	NA	NA	NA	NA	-0.016	3.94E-06	1.27E-02	-0.012
cg11096905	17	63535567	*AXIN2*	NA	NA	NA	NA	-0.016	4.15E-06	1.32E-02	-0.015
cg24698536	12	109250515	*SSH1*	NA	NA	NA	NA	-0.026	4.22E-06	1.34E-02	-0.022
cg24239148	10	63422841	*C10orf107*	NA	NA	NA	NA	0.007	4.69E-06	1.47E-02	0.007
cg10673740	10	121411826	*BAG3*	NA	NA	NA	NA	0.019	5.08E-06	1.58E-02	0.021
cg22057874	2	179184866	*OSBPL6*	NA	NA	NA	NA	0.013	5.25E-06	1.62E-02	0.013
cg04640109	8	106330173	*ZFPM2*	NA	NA	NA	NA	0.011	5.43E-06	1.66E-02	0.013
cg12492906	12	109250545	*SSH1*	NA	NA	NA	NA	-0.020	5.87E-06	1.78E-02	-0.020
cg11641006	2	235213874	*RPS20P12*	NA	NA	NA	NA	0.016	5.92E-06	1.78E-02	0.014
cg11868728	20	22568116	*FOXA2*	NA	NA	NA	NA	0.009	6.03E-06	1.80E-02	0.007
cg16517298	1	230413174	*GALNT2*	NA	J	NA	NA	-0.027	6.09E-06	1.80E-02	-0.027
cg05593775	10	102778743	*PDZD7*	NA	NA	NA	NA	0.016	6.18E-06	1.81E-02	0.022
cg03354508	6	147769498	*YAP1P1*	NA	NA	NA	NA	0.011	6.85E-06	1.98E-02	0.010
cg24173182	17	1961286	*HIC1*	NA	NA	NA	Z	0.022	6.86E-06	1.98E-02	0.024
cg13784312	9	134609065	*RAPGEF1*	NA	NA	NA	M	-0.016	7.22E-06	2.06E-02	-0.020
cg26554302	15	22467326	*IGHV1OR15-3*	NA	NA	NA	NA	0.014	7.27E-06	2.06E-02	0.020
cg22043296	11	1892139	*LSP1*	NA	NA	NA	NA	-0.012	7.42E-06	2.09E-02	-0.016
cg04800195	5	180287904	*ZFP62*	NA	NA	NA	NA	0.003	7.56E-06	2.11E-02	0.003
cg07249149	7	1035363	*C7orf50*	NA	NA	NA	NA	-0.013	7.62E-06	2.11E-02	-0.015
cg15963463	1	25253237	*RUNX3*	NA	NA	NA	NA	-0.011	7.69E-06	2.11E-02	-0.008
cg14086013	6	41604740	*MDFI*	NA	NA	NA	NA	0.012	7.74E-06	2.11E-02	0.015
cg02010738	19	2095466	*MOB3A*	NA	NA	NA	Z	-0.020	8.13E-06	2.20E-02	-0.019
cg18794473	8	77617694	*ZFHX4*	NA	NA	NA	NA	0.004	8.61E-06	2.30E-02	0.003
cg26804595	16	749581	*FBXL16*	NA	NA	NA	NA	-0.010	8.61E-06	2.30E-02	-0.008
cg14027333	6	32116317	*PRRT1*	NA	NA	NA	NA	-0.005	8.76E-06	2.32E-02	-0.004
cg02356218	11	124352048	*OR8B9P*	NA	NA	NA	NA	-0.014	9.03E-06	2.37E-02	-0.013

Probe	Chr	Basepair[b]	Nearest gene	Newborn[c]: Site	Newborn[c]: Gene	Adult[c]: Site	Adult[c]: Gene	Model1[d] Coeff	Model1[d] *P*-value	Model1[d] *q*-value	Δ β-value[e]
cg14349977	4	187219430	*LOC285441*	NA	NA	NA	NA	-0.022	9.09E-06	2.37E-02	-0.021
cg09063262	11	85358506	*TMEM126A*	NA	NA	NA	NA	-0.022	9.37E-06	2.41E-02	-0.021
cg26441486	22	50317300	*CRELD2*	NA	NA	NA	NA	-0.018	9.38E-06	2.41E-02	-0.019
cg00029284	12	111731203	*CUX2*	J	J	NA	NA	-0.009	9.62E-06	2.46E-02	-0.009
cg12160087	12	120531986	*CCDC64*	NA	NA	NA	NA	-0.010	1.14E-05	2.88E-02	-0.010
cg19727396	1	230415185	*GALNT2*	NA	J	NA	NA	-0.018	1.15E-05	2.88E-02	-0.020
cg06949812	17	38517993	*GJD3*	NA	NA	NA	NA	0.013	1.20E-05	3.00E-02	0.015
cg26103179	12	119591664	*SRRM4*	NA	NA	NA	NA	-0.015	1.32E-05	3.27E-02	-0.012
cg09488203	10	95327884	*O3FAR1*	NA	NA	NA	NA	0.009	1.33E-05	3.27E-02	0.009
cg13928411	16	22103791	*VWA3A*	NA	NA	NA	NA	0.009	1.35E-05	3.29E-02	0.008
cg16231923	15	73923634	*NPTN*	NA	NA	NA	NA	0.020	1.36E-05	3.30E-02	0.023
cg20274009	20	32450846	*TPM3P2*	NA	NA	NA	NA	0.007	1.37E-05	3.32E-02	0.004
cg22877366	1	12107668	*MIIP*	NA	NA	NA	NA	-0.020	1.40E-05	3.35E-02	-0.019
cg16449012	4	17781880	*FAM184B*	NA	NA	NA	NA	0.011	1.42E-05	3.39E-02	0.011
cg00433296	8	22223852	*SLC39A14*	NA	NA	NA	NA	0.010	1.43E-05	3.39E-02	0.010
cg05656688	1	25254088	*RUNX3*	NA	NA	NA	NA	-0.014	1.44E-05	3.40E-02	-0.015
cg08142858	1	2718742	*TTC34*	NA	NA	NA	NA	0.008	1.49E-05	3.49E-02	0.005
cg25415695	5	134828036	*TIFAB*	NA	NA	NA	NA	0.012	1.61E-05	3.70E-02	0.012
cg25122233	12	54412506	*HOXC4*	NA	NA	NA	NA	0.020	1.61E-05	3.70E-02	0.018
cg18828927	6	29275263	*OR14J1*	NA	NA	NA	NA	0.008	1.62E-05	3.70E-02	0.006
cg08698721	14	101294147	*MEG3*	J	J	NA	NA	0.013	1.66E-05	3.74E-02	0.012
cg22879098	5	672845	*TPPP*	NA	NA	NA	NA	-0.030	1.67E-05	3.74E-02	-0.025
cg07448928	5	427755	*AHRR*	NA	J	NA	Z; S; M	0.007	1.67E-05	3.74E-02	0.006
cg12443001	1	2467281	*HES5*	NA	NA	NA	NA	0.010	1.67E-05	3.74E-02	0.008
cg23458168	19	30864867	*ZNF536*	NA	NA	NA	NA	0.029	1.76E-05	3.90E-02	0.033
cg13547053	2	179184864	*OSBPL6*	NA	NA	NA	NA	0.010	1.83E-05	3.99E-02	0.011
cg17345450	6	166259938	*SDIM1*	NA	NA	NA	NA	-0.011	1.83E-05	3.99E-02	-0.014
cg23928512	17	79970192	*ASPSCR1*	NA	NA	NA	NA	-0.011	1.83E-05	3.99E-02	-0.013
cg07553761	3	160167977	*TRIM59*	NA	NA	NA	NA	0.004	1.94E-05	4.19E-02	0.005
cg13459104	9	98849721	*LOC158435*	NA	NA	NA	NA	0.003	1.95E-05	4.19E-02	0.003
cg15893360	12	120700361	*PXN*	NA	NA	NA	NA	-0.013	1.96E-05	4.19E-02	-0.012
cg02404974	6	41376295	*LOC100505711*	NA	NA	NA	NA	0.015	1.99E-05	4.23E-02	0.015
cg04807108	15	78697933	*RWDD1P1*	NA	NA	NA	NA	0.022	2.01E-05	4.24E-02	0.026
cg22171758	12	54146133	*CALCOCO1*	NA	NA	NA	NA	0.012	2.02E-05	4.24E-02	0.010
cg03486379	10	34391256	*PARD3*	NA	NA	NA	NA	0.009	2.03E-05	4.24E-02	0.008

Probe	Chr	Basepair[b]	Nearest gene	Newborn[c]: Site	Newborn[c]: Gene	Adult[c]: Site	Adult[c]: Gene	Model1[d] Coeff	Model1[d] *P*-value	Model1[d] *q*-value	Δ β-value[e]
cg07464358	7	100952364	*RABL5*	NA	NA	NA	NA	0.008	2.08E-05	4.30E-02	0.011
cg19675142	1	116107015	*LOC100132332*	NA	NA	NA	NA	0.013	2.08E-05	4.30E-02	0.013
cg08755703	16	54394989	*IRX3*	NA	NA	NA	NA	0.017	2.12E-05	4.36E-02	0.022
cg20174893	7	97600926	*MGC72080*	NA	NA	NA	NA	-0.010	2.16E-05	4.41E-02	-0.009
cg10788371	11	76381040	*LRRC32*	NA	NA	NA	NA	-0.010	2.20E-05	4.47E-02	-0.009
cg06509837	19	610955	*HCN2*	NA	NA	NA	NA	-0.026	2.22E-05	4.47E-02	-0.025
cg18209323	7	92672878	*SAMD9*	NA	NA	NA	NA	0.016	2.23E-05	4.47E-02	0.016
cg11064524	8	118875129	*EXT1*	NA	J	NA	NA	-0.011	2.33E-05	4.65E-02	-0.013
cg03687532	16	54228358	*FTO*	NA	J	NA	NA	0.012	2.41E-05	4.77E-02	0.015
cg26580869	19	611506	*HCN2*	NA	NA	NA	NA	-0.019	2.41E-05	4.77E-02	-0.020
cg13455434	8	30243930	*RBPMS*	NA	NA	NA	NA	0.026	2.44E-05	4.77E-02	0.027
cg16944958	6	170403962	*LOC100505903*	NA	NA	NA	NA	-0.012	2.44E-05	4.77E-02	-0.010
cg26827653	11	125366540	*MGC39545*	NA	NA	NA	NA	0.012	2.50E-05	4.85E-02	0.012
cg19563365	11	86508399	*PRSS23*	NA	NA	NA	Z	0.007	2.56E-05	4.94E-02	0.007

Abbreviations: Chr: chromosome, coeff: coefficient, NA: not applicable

[a]FDR q<0.05 (Model1). [b]Physical location in basepairs (Human genome build GRCh37/hg19). [c]Replication at the site and/or gene level using previously published studies that utilized Illumina HumanMethylation450 BeadChips. Study abbreviations: J: Joubert, BR et al. 2012, M: Monick, MM et al. 2012, S: Shenker, NS et al. 2013, and Z: Zeilinger, S et al. 2013. [d]Model1: Methylation β-value = maternal smoking + infant's sex + infant's cleft status + batch effect + bisulfite conversion efficiency. [e]β-value represents the ratio of methylated signal to total signal (methylated plus unmethylated). This shows the difference between the mean β-value of smokers and non-smokers.

[*]Meets bonferroni correction ($p<1.4x10^{-7}$).

Table S9. Technical replicate assessment.[a]

Data processing	Mean Pearson correlation (uncentered)	Mean Pearson correlation (mean centered)[b]	Mean of squared difference
**Replicates**			
Raw data	0.993	0.573	0.0021
Normalized data[c]	0.996	0.692	0.0014
Normalized plus adjustments[d]	0.998	0.692	0.0007
**Non-replicates[e]**			
Raw data	0.983	-0.041	0.0024
Normalized data[c]	0.986	-0.043	0.0023
Normalized plus adjustments[d]	0.993	-0.044	0.0017

[a]20 duplicate pairs ($N_{total} = 40$). [b]For each CpG probe, the mean β-value was calculated across the 40 technical replicates and then subtracted from each β-value. The Pearson correlation was then calculated for each of the 20 duplicate pairs; the mean Pearson correlation across all pairs is reported in the table. [c]Corrected for dye bias, background corrected, and quantile normalized. [d]Also adjusted for batch effects (96-well plate) and bisulfite conversion efficiency. [e]Non-replicate pairs were created from the 40 technical replicates. All pairwise combinations were considered, except for those that were duplicates.

Supplemental figures

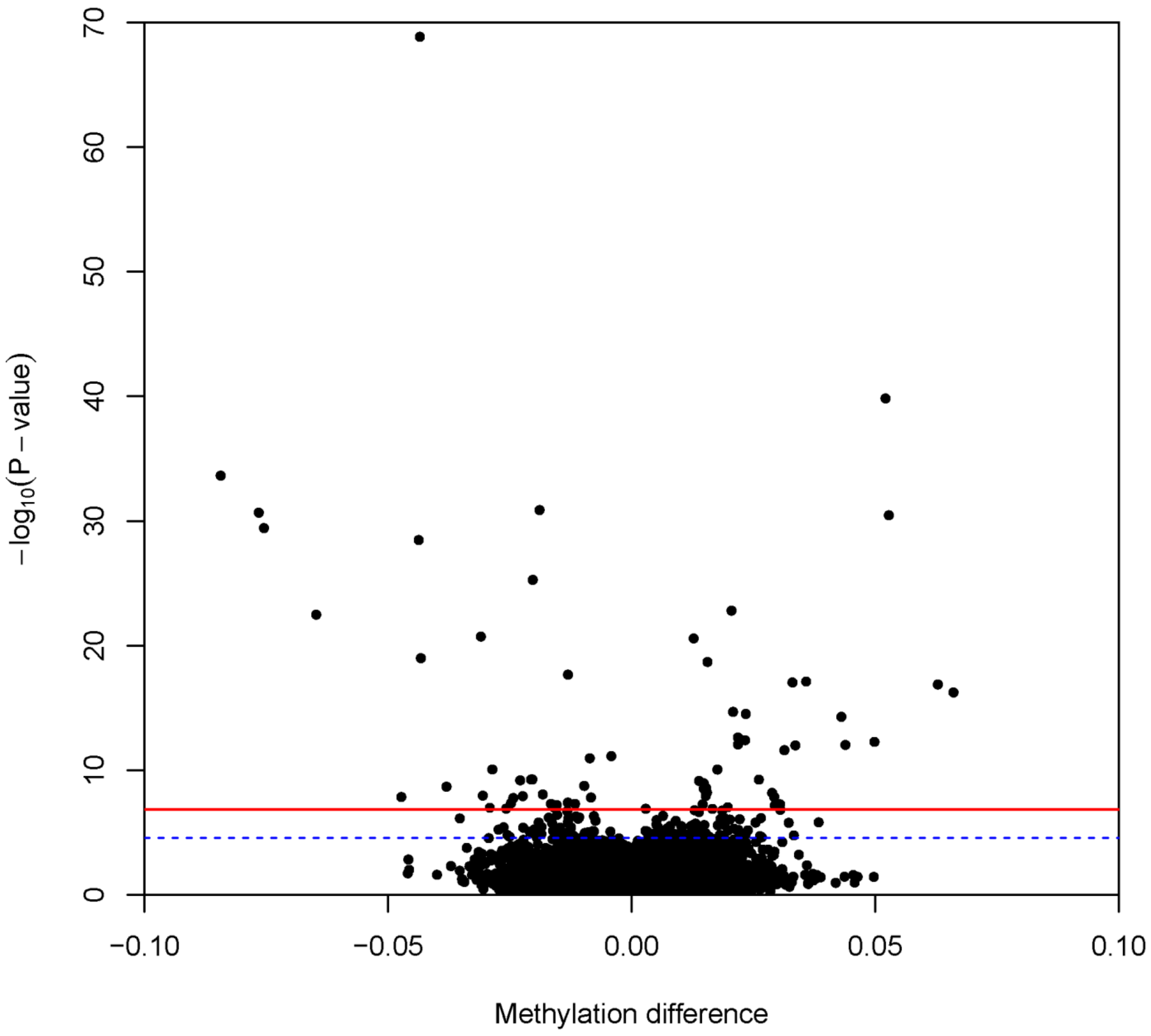

Figure S1. Volcano plot showing the methylation difference between infants of smokers and infants of non-smokers (Mean β_{cases} – Mean $\beta_{controls}$) against the $-\log_{10}$(P-value) based on results from Model1. The blue dashed horizontal line marks genome-wide significance using FDR $q<0.05$ as a cutoff ($p < 2.69 \times 10^{-5}$), while the red solid horizontal line marks genome-wide significance using a bonferroni correction ($p<1.40 \times 10^{-7}$).

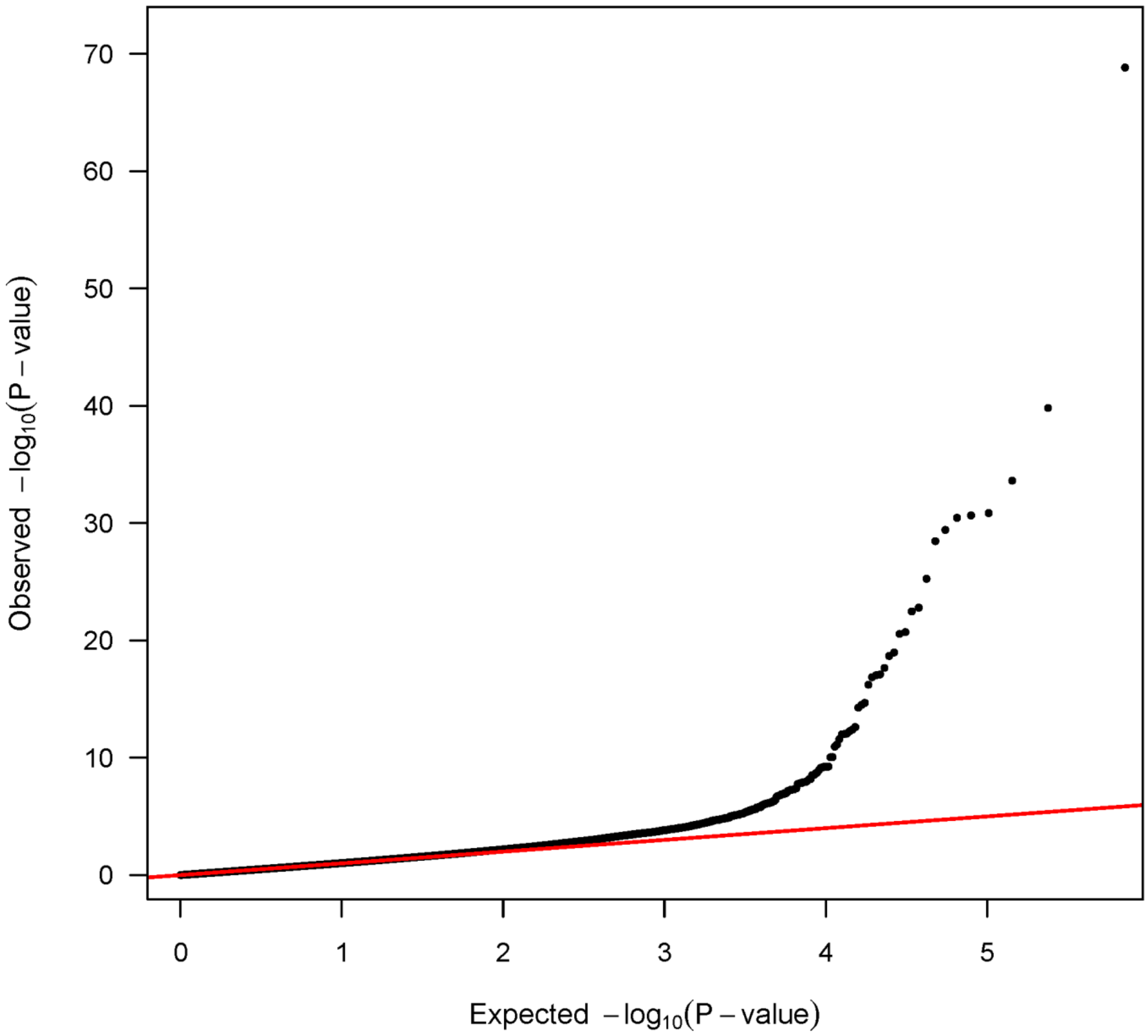

Figure S2. Quantile-quantile (Q-Q) plot showing the observed (Model1) versus expected -$\log_{10}$(P-values) under the null hypothesis of no association.

a)

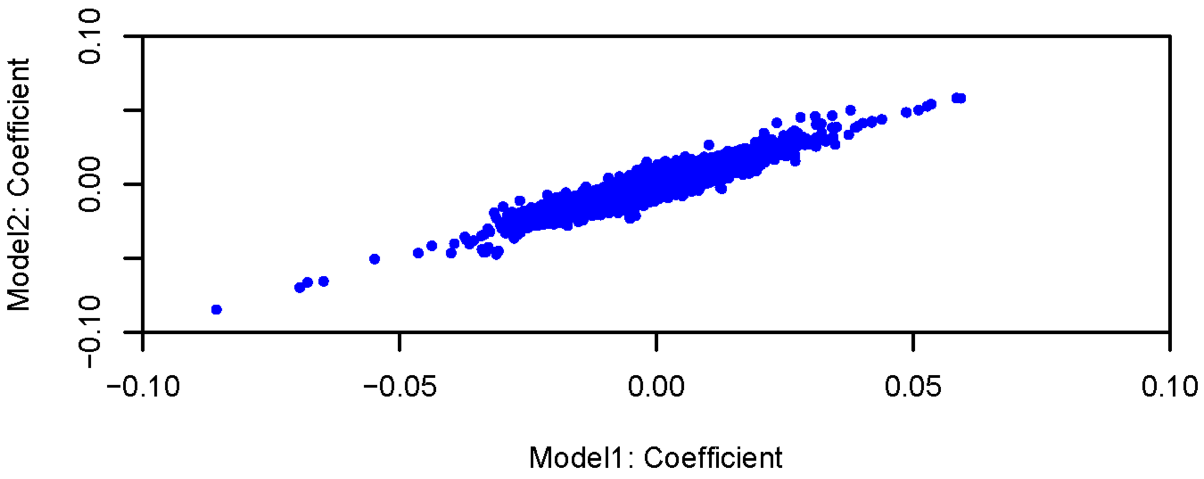

b)

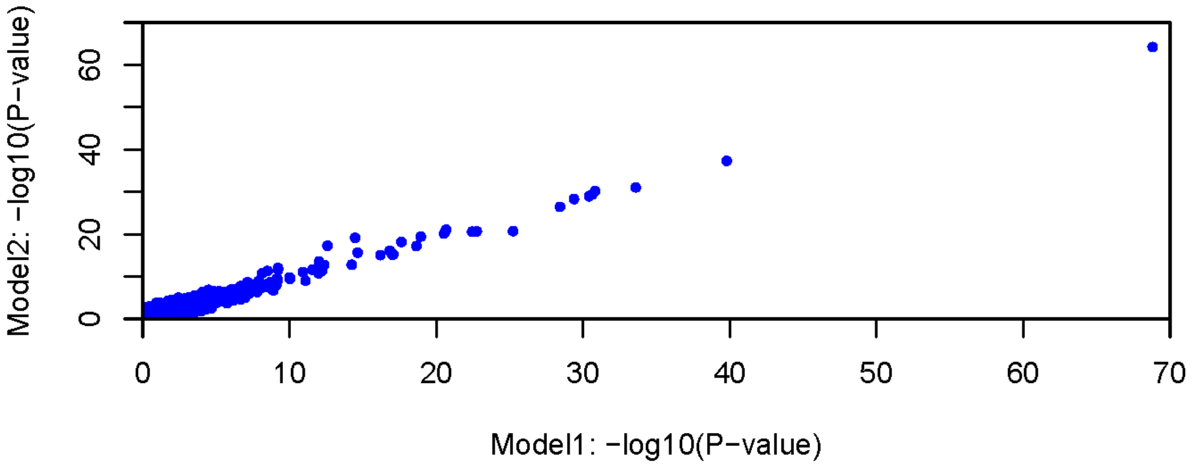

Figure S3. Comparison of results from Model1 and Model2. Model 1: Methylation β-value = maternal smoking + infant's sex + infant's cleft status + batch effect + bisulfite conversion efficiency; Model2: Methylation β-value = maternal smoking + infant's sex + infant's cleft status + batch effect + bisulfite conversion efficiency + maternal alcohol use + maternal education + maternal age at delivery + maternal folic acid supplement use + infant's birth weight + adjustment for 5 different cell type proportions. A) Comparison of the smoking coefficient in Model1 versus Model2 (Pearson correlation=0.93), B) Comparison of the smoking $-\log_{10}$(P-value) in Model1 versus Model2 (Pearson correlation= 0.88).

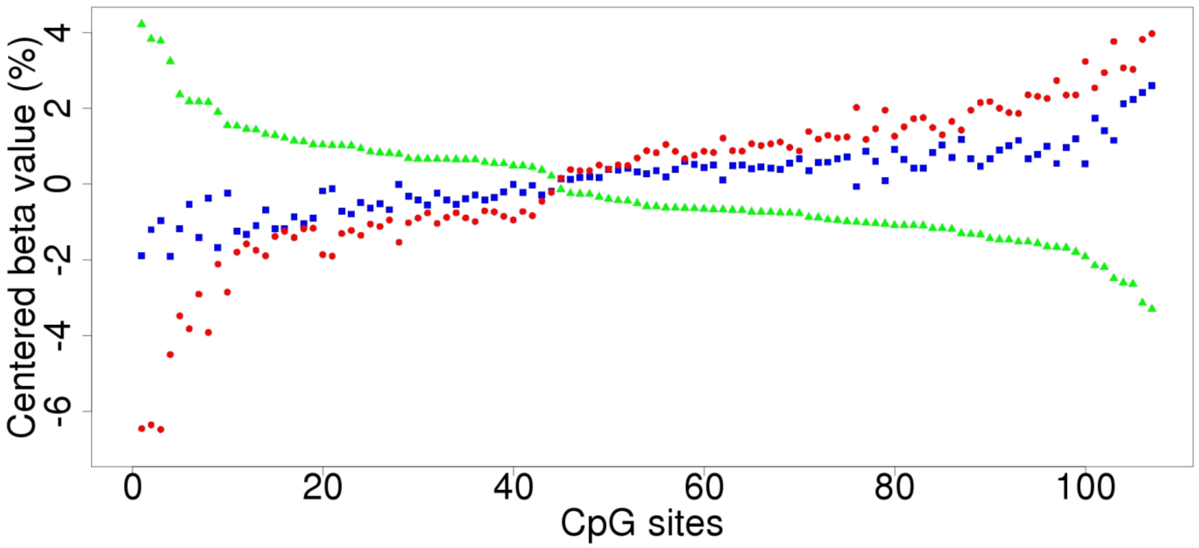

Figure S4. Genome-wide significant CpG sites that showed a dose-response relationship (N=107/185). Three exposure classes are shown: 1) Non-active smokers (green triangles), 2) light smokers (blue squares), and 3) moderate/heavy smokers (red circles).

MEG3

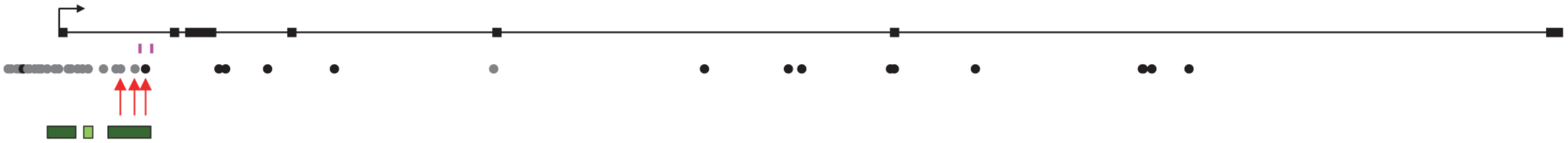

CpG sites

● 30%-70% methylated

● 70%-100% methylated

Figure S5. MEG3 gene diagram (GenBank accession number, NR_002766.2**)**. The pink rectangles mark the regions with putative CTCF binding sites as determined by Wylie, AA et al. 2000. The red arrows point to the three CpG sites we found that showed increased methylation ($p<0.0002$) in newborns of women who smoked during pregnancy. The green rectangles mark the CpG islands (GC content ≥ 50%, length > 200 bp with islands < 300 bp shown in light green, Observed/Expected CpGs > 0.6). The average methylation observed in the CpG sites colored grey is 57% and the average methylation observed in the CpG sites colored black is 88%.

References

Bolstad BM, Irizarry RA, Astrand M, Speed TP. 2003. A comparison of normalization methods for high density oligonucleotide array data based on variance and bias. Bioinformatics 19:185-193.

Davis S, Du P, Bilke S, Triche T, Bootwalla M. 2013. methylumi: Handle Illumina methylation data. R package version 2.6.1.

Irizarry RA, Hobbs B, Collin F, Beazer-Barclay YD, Antonellis KJ, Scherf U, et al. 2003. Exploration, normalization, and summaries of high density oligonucleotide array probe level data. Biostatistics 4:249-264.

Joubert BR, Haberg SE, Nilsen RM, Wang X, Vollset SE, Murphy SK, et al. 2012. 450K epigenome-wide scan identifies differential DNA methylation in newborns related to maternal smoking during pregnancy. Environ Health Perspect 120:1425-1431.

Monick MM, Beach SR, Plume J, Sears R, Gerrard M, Brody GH, et al. 2012. Coordinated changes in AHRR methylation in lymphoblasts and pulmonary macrophages from smokers. Am J Med Genet B Neuropsychiatr Genet 159B:141-151.

Price ME, Cotton AM, Lam LL, Farre P, Emberly E, Brown CJ, et al. 2013. Additional annotation enhances potential for biologically-relevant analysis of the Illumina Infinium HumanMethylation450 Beadchip array. Epigenetics Chromatin 6:4-19.

Shenker NS, Polidoro S, van Veldhoven K, Sacerdote C, Ricceri F, Birrell MA, et al. 2012. Epigenome-wide association study in the European Prospective Investigation into Cancer and Nutrition (EPIC-Turin) identifies novel genetic loci associated with smoking. Hum Mol Genet 22:843-851.

Trevor H, Tibshirani R, Narasimhan B, Chu G. 2014. Impute: Imputation for microarray data. R package version 1.34.0.

Wylie AA, Murphy SK, Orton TC, Jirtle RL. 2000. Novel imprinted DLK1/GTL2 domain on human chromosome 14 contains motifs that mimic those implicated in IGF2/H19 regulation. Genome Res 10:1711-1718.

Zeilinger S, Kuhnel B, Klopp N, Baurecht H, Kleinschmidt A, Gieger C, et al. 2013. Tobacco smoking leads to extensive genome-wide changes in DNA methylation. PLoS One 8:e63812.
